# Fha Interaction with Phosphothreonine of TssL Activates Type VI Secretion in *Agrobacterium tumefaciens*

**DOI:** 10.1371/journal.ppat.1003991

**Published:** 2014-03-13

**Authors:** Jer-Sheng Lin, Hsin-Hui Wu, Pang-Hung Hsu, Lay-Sun Ma, Yin-Yuin Pang, Ming-Daw Tsai, Erh-Min Lai

**Affiliations:** 1 Institute of Plant and Microbial Biology, Academia Sinica, Taipei, Taiwan; 2 Institute of Biological Chemistry, Academia Sinica, Taipei, Taiwan; 3 Structural Biology Program, National Tsing Hua University, Hsinchu, Taiwan; 4 Institute of Bioinformatics and Structural Biology, National Tsing Hua University, Hsinchu, Taiwan; 5 Genomics Research Center, Academia Sinica, Taipei, Taiwan; 6 Department of Life Science, Institute of Bioscience and Biotechnology, National Taiwan Ocean University, Keelung, Taiwan; 7 Institute of Biochemical Sciences, National Taiwan University, Taipei, Taiwan; University of Washington, United States of America

## Abstract

The type VI secretion system (T6SS) is a widespread protein secretion system found in many Gram-negative bacteria. T6SSs are highly regulated by various regulatory systems at multiple levels, including post-translational regulation via threonine (Thr) phosphorylation. The Ser/Thr protein kinase PpkA is responsible for this Thr phosphorylation regulation, and the forkhead-associated (FHA) domain-containing Fha-family protein is the sole T6SS phosphorylation substrate identified to date. Here we discovered that TssL, the T6SS inner-membrane core component, is phosphorylated and the phosphorylated TssL (*p-*TssL) activates type VI subassembly and secretion in a plant pathogenic bacterium, *Agrobacterium tumefaciens*. Combining genetic and biochemical approaches, we demonstrate that TssL is phosphorylated at Thr 14 in a PpkA-dependent manner. Further analysis revealed that the PpkA kinase activity is responsible for the Thr 14 phosphorylation, which is critical for the secretion of the T6SS hallmark protein Hcp and the putative toxin effector Atu4347. TssL phosphorylation is not required for the formation of the TssM-TssL inner-membrane complex but is critical for TssM conformational change and binding to Hcp and Atu4347. Importantly, Fha specifically interacts with phosphothreonine of TssL via its pThr-binding motif *in vivo* and *in vitro* and this interaction is crucial for TssL interaction with Hcp and Atu4347 and activation of type VI secretion. In contrast, pThr-binding ability of Fha is dispensable for TssM structural transition. In conclusion, we discover a novel Thr phosphorylation event, in which PpkA phosphorylates TssL to activate type VI secretion via its direct binding to Fha in *A. tumefaciens*. A model depicting an ordered TssL phosphorylation-induced T6SS assembly pathway is proposed.

## Author Summary

The bacterial type VI secretion system (T6SS) resembles a contractile phage tail structure and functions to deliver effectors to eukaryotic or prokaryotic target cells for the survival of many pathogenic bacteria. T6SS is highly regulated by various regulatory systems at multiple levels in response to environmental cues. Post-translational regulation via threonine (Thr) phosphorylation is an emerging theme in regulating prokaryotic signaling, including T6SS; the knowledge is mainly contributed by studies of Hcp secretion island 1-encoded T6SS (H1-T6SS) of *Pseudomonas aeruginosa*. Here, we discover a new phosphorylated target, a T6SS core-component TssL, and demonstrate that this Thr phosphorylation event post-translationally regulates type VI secretion in a plant pathogenic bacterium, *Agrobacterium tumefaciens*. We provide the first demonstration that the specific binding of Fha, a forkhead-associated domain-containing protein, to the phosphorylated target is required to stimulate type VI secretion. Genetic and biochemical data strongly suggest an ordered TssL-phosphorylation–dependent assembly and secretion pathway.

## Introduction

The type VI secretion system (T6SS) is the most recently described protein secretion system encoded as one or multiple copies in ∼25% of all sequenced Gram-negative bacteria [1]. T6SS is highly regulated and exhibits cytotoxicity to eukaryotic or bacterial hosts in a contact-dependent manner [2]–[6]. Growing evidence of the T6SS structure and imaging analyses documented that T6SS assembles into a contractile phage tail-like structure consisting of a TssB/TssC tubule structure [7]–[9], which is proposed to wrap around the Hcp tail tube and function to propel Hcp and effector secretion [10]. In support of T6SS functioning as a contractile phage tail-like structure, Hcp and VgrG are also detected on the cell surface and directly interact with each other and the TssB/TssC tubule [11]. Importantly, Hcp can interact with known or putative secreted effectors [11]–[13] and was recently identified to function as a chaperone and receptor of secreted substrates [12]. Time-lapse fluorescent microscopy further allowed for visualizing the dynamic T6SS activity that occurs between pairs of interacting cells, termed “T6SS dueling” [14], and killing activity at single-cell levels [15]–[17].

Bioinformatics and mutagenesis analyses revealed that T6SS consists of approximately 13–14 conserved components of type VI secretion (Tss, nomenclature proposed by Shalom et al.) [18] required for type VI secretion [10], [11], [13], [19]. Among them, TssM and TssL are the core inner-membrane proteins forming a stable inner-membrane complex connecting an outer-membrane complex [13], [20], [21]. TssM exhibits ATPase activity, and ATP binding-induced conformational change and subsequent ATP hydrolysis are important for the recruitment of Hcp to the TssM-TssL complex likely by directly interacting with the periplasmic domain of TssL [22]. The crystal structure of the N-terminal cytoplasmic domain of TssL has been reported [23], [24]. The cytoplasmic domain of TssL forms dimers, and this self-interaction is required for its function in enteroaggregative *Escherichia coli* Sci-1 T6SS [23]. Structural analysis of *Francisella novicida* cytoplasmic TssL revealed a surface-exposed groove that may represent a functional site for T6SS function [24] and is proposed to serve as a cytosolic hook to recruit the secreted substrates to the T6SS [25].

Interestingly, a subset of T6SS gene clusters also encode orthologs of Ser/Thr protein kinase (PpkA), phosphatase (PppA), and forkhead-associated (FHA) domain-containing proteins that are involved in regulating the Thr phosphorylation event [1], [26]–[28]. To date, our knowledge of the Thr phosphorylation regulatory mechanism is mostly due to several excellent studies in *P. aeruginosa*, in which the Hcp secretion island 1-encoded T6SS (H1-T6SS) is regulated positively by PpkA and negatively by the cognate phosphatase PppA via Thr phosphorylation on an FHA domain-containing protein, Fha1 [29]. A recent phosphoproteomic study also revealed that PpkA-dependent phosphorylation of Fha is required for T6SS activation in *Serratia marcescens* [28]. In addition, type VI secretion associated genes Q, R, S, T (TagQRST) function upstream of PpkA to promote kinase activity and subsequent type VI secretion in *P. aeruginosa* [30], [31]. TagF was identified as the repressor, whose absence caused the activation of a Thr phosphorylation-independent T6SS pathway [27]. Interestingly, Thr phosphorylation-dependent type VI activation could be stimulated by a cue during *P. aeruginosa* surface growth [27]. Also, PpkA, PppA, and TagT were required for the activation of the *P. aeruginosa* T6SS dueling activity when encountering the T6SS attack via contact with *Vibrio cholerae* cells [17]. Thus, the Thr phosphorylation-positive regulator TagQRST localized in the membranes [30], [31] may function to perceive and transduce signals to PpkA for activation of the local T6SS assembly for counterattack [17].

Although quite common in eukaryotes, this Ser/Thr phosphorylation regulatory mechanism is an emerging theme in prokaryotic signaling [32]. The FHA domain is a specific phosphothreonine (pThr) recognition unit [33]–[35] that was first identified as a conserved region via sequence analysis in a subset of forkhead-type transcription factors [36]. Numerous studies revealed that FHA domain-containing proteins play critical roles in diverse cellular processes in eukaryotes [35] and in a few prokaryotes, with a wealth of information obtained from *Mycobacterium tuberculosis* encoding 11 Ser/Thr protein kinases and 7 FHA domains in 6 proteins [37]–[39]. The importance of the FHA domain in activating type VI secretion and activity was also clearly demonstrated in the H1-T6SS of *P. aeruginosa* [29], [30]. Fha1 focally co-localized with ClpV1, an AAA+ ATPase specifically binding to the contracted TssB/TssC tubule for disassembly and cycling [7]–[9]. Importantly, Fha1 is required for ClpV1 focal recruitment, which suggests that Fha1 is a core scaffold protein of the H1-T6SS [29]. Interestingly, PpkA is crucial for both Fha1 and ClpV1 focal localization [29] and ClpV1 dynamics [17], but Fha1 phosphorylation is not required for ClpV1 recruitment but is critical for Hcp1 secretion [30]. Thus, PpkA may phosphorylate additional T6SS component(s), whereby its phosphorylation status may be critical for Fha and ClpV focal recruitment and subsequent type VI secretion. However, no additional T6SS protein has been identified as the PpkA target to date.

In this study, we investigated the involvement of Thr phosphorylation in regulating type VI secretion in *Agrobacterium tumefaciens*, a soil phytopathogen capable of causing crown gall disease in wide range of plants. We discovered that TssL, a T6SS core component of the inner-membrane complex [20], is phosphorylated in a PpkA-dependent manner when *A. tumefaciens* is transcriptionally activated by the ExoR-ChvG/ChvI signaling cascade upon sensing the acidic signal [40]. We demonstrated that TssL is phosphorylated at Thr 14 and that this phosphorylation is crucial for stimulating secretion of the type VI hallmark protein Hcp and Atu4347, a putative toxin effector homologous to the anti-bacterial toxin secreted small protein (Ssp) in *S. marcescens* [6], [11]. Remarkably, TssL phosphorylation triggered TssM conformational change and promoted its binding to Hcp and Atu4347. The pThr binding ability of Fha is critical for specific binding to phosphorylated TssL (*p-*TssL) and this specific *p-*TssL-Fha interaction is required for efficient binding of Hcp and Atu4347 to *p-*TssL and activation of type VI secretion. We proposed that PpkA kinase initiates the complex assembly by phosphorylating TssL to increase the ATP binding ability of TssM ATPase for energy production and leads to the recruitment of Fha for activation of type VI secretion.

## Results

### PpkA- and Fha-dependent post-translational regulation of Hcp secretion from *A. tumefaciens*

**Figure 1:**
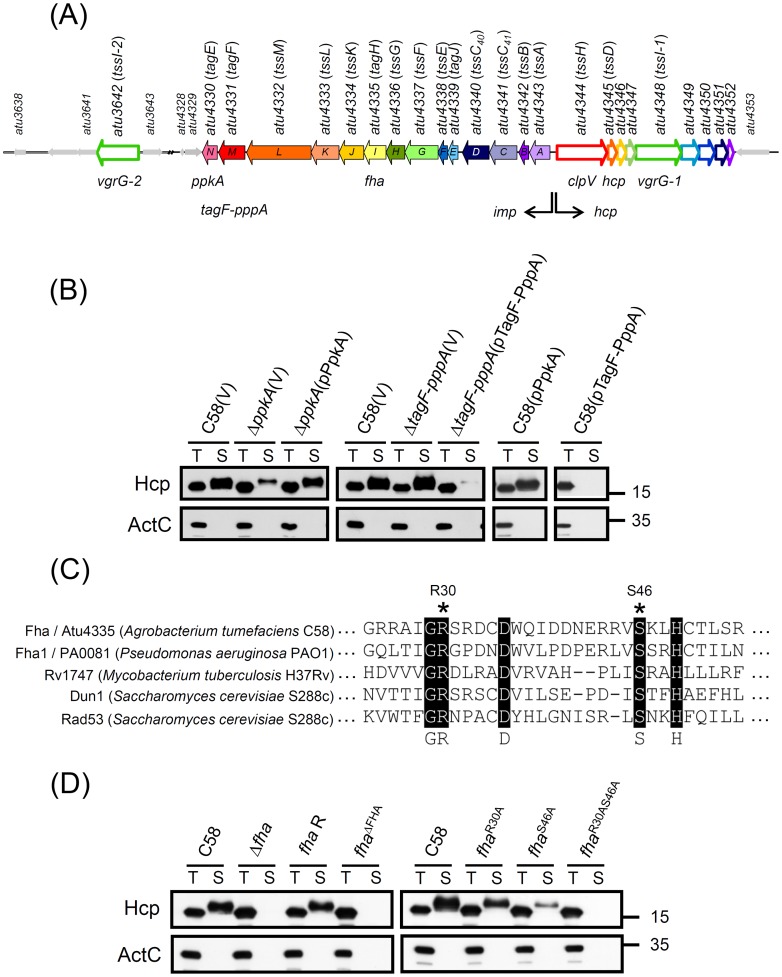
PpkA- and Fha-dependent post-translational regulation of Hcp secretion from *A. tumefaciens.* (**A**) The *imp* operon encoding 14 genes (*atu4343* to *atu4330* or *impA-N*) and the *hcp* operon encoding 9 genes (*atu4344* to *atu4352*) and *atu3642* (*vgrG-2*) encoded by *A. tumefaciens* strain C58 was designated *tss* or *tag* based on nomenclature proposed by Shalom et al. (2007) [18] and common names *ppkA*, *tagF-pppA*, and *fha*. This schematic was from Lin et al. (2013) [11]. (**B**) Hcp secretion analysis. Western blot analysis of total (T) and secreted (S) proteins isolated from wild-type C58, Δ*ppkA*, and Δ*tagF-pppA* mutants harboring the vector pRL662 (V) or *ppkA* complemented plasmid (pPpkA) or *tagF-pppA* complemented plasmid (pTagF-PppA) with specific antibodies. (**C**) The amino acid sequence alignment of the FHA domain of Fha (Atu4335) and selected Fha-family proteins indicating the conserved pThr binding motif. Conserved amino acid residues are highlighted in black and marked below, and R30 and S46 used for mutagenesis are indicated with an asterisk. Sequences were aligned and highlighted by use of ClustalW2 (http://www.ebi.ac.uk/Tools/msa/clustalw2/). (**D**) Hcp secretion assay for chromosomally encoded *fha* variants, including *fha* deletion (Δ*fha*), *fha* wild-type revertant with double crossover (*fha* R), *fha* with deletion of the entire FHA domain from 25 to 76 aa (*fha* ^ΔFHA^), and *fha* with substitutions of pThr binding motif (*fha* ^R30A^, *fha* ^S46A^, and *fha* ^R30AS46A^). For secretion assays in (B) and (D), the non-secreted protein ActC was an internal control. The proteins analyzed and sizes of molecular weight standards are on the left and right, respectively.

The presence of genes encoding the Fha-family protein, PpkA kinase, and PppA phosphatase in a subset of T6SSs suggested the involvement of a Thr phosphorylation regulatory pathway in these bacteria [1], [26], [27]. However, this Thr phosphorylation regulation of T6SS has only been demonstrated in H1-T6SS of *P. aeruginosa* [29] and *S. marcescens* [28]. Bioinformatic analysis revealed the homology of Atu4330 to PpkA, Atu4331 with N-terminal domain and C-terminal domain to TagF and PppA respectively (thus renamed as TagF-PppA), and Atu4335 to Fha encoded in the T6SS gene cluster of *A. tumefaciens* strain C58 (Figure 1A) [11], [26], [29], [32], [41]. We previously identified that Hcp secretion was abolished in Δ*fha* and its abundance was greatly reduced in Δ*ppkA*, whereas Hcp secretion level was not significantly altered with the deletion of *tagF-pppA* (*atu4331*) [11]. These data suggested that the Thr phosphorylation pathway (TPP) may play a role to regulate type VI secretion in *A. tumefaciens*, but the molecular mechanisms underlying this potential Thr phosphorylation regulation remain unknown.

Thus, we first performed complementation tests and overexpression analysis to determine the role of PpkA and TagF-PppA in regulating Hcp secretion. The reduced Hcp secretion in Δ*ppkA* could be fully restored by *trans* complementation (Figure 1B). In contrast, Hcp secretion was abolished when TagF-PppA was overexpressed on plasmids in both C58 and Δ*tagF-pppA* strains (Figure 1B). The negative effect of overexpressed TagF-PppA in Hcp secretion was specific because overexpressed PpkA in C58 did not affect Hcp secretion levels. Therefore, PpkA positively and TagF-PppA negatively regulated Hcp secretion in *A. tumefaciens*.

**Figure 2:**
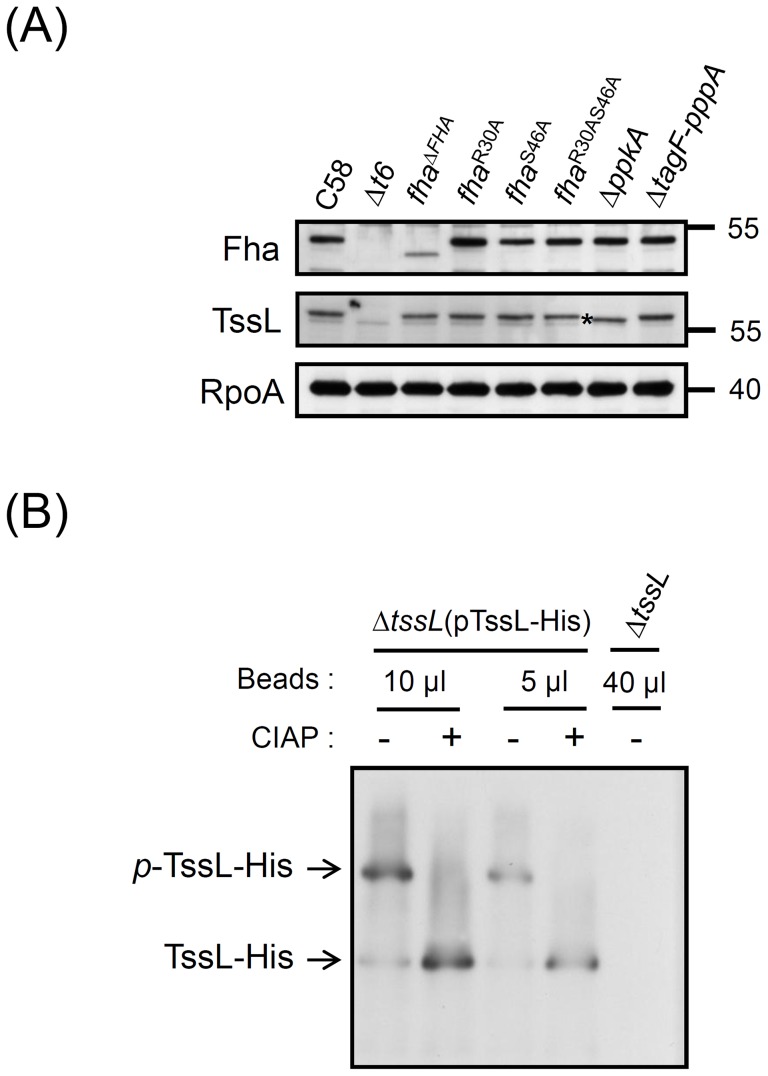
TssL phosphorylation analysis by Phos-tag. (**A**) Western blot analysis with regular SDS-PAGE of total proteins isolated from various *A. tumefaciens* strains examined with specific antibodies. RNA polymerase α subunit RpoA was an internal control. The TssL protein band with faster migration in Δ*ppkA* mutant is marked with an asterisk. (**B**) Phos-tag SDS-PAGE analysis. Different volumes of Ni-NTA resins containing TssL-His were treated with (+) or without (−) calf intestinal alkaline phosphatase (CIAP). Western blot analysis of proteins were separated by 7% Phos-tag SDS-PAGE and examined by specific antibody against 6×His. Total proteins from Δ*tssL* mutant were a negative control. Phos-tag SDS-PAGE revealed the upper band indicating phosphorylated TssL-His (*p*-TssL-His) and lower band indicating unphosphorylated TssL-His.

In *P. aeruginosa*, PpkA phosphorylates Fha1; both its phosphorylation status and FHA domain responsible for pThr binding ability are critical for Hcp1 secretion [29]. In *A. tumefaciens*, Fha contains a putative N-terminal FHA domain with amino acid residues (R30 and S46) corresponding to the conserved pThr binding residues [33], [35] (Figure 1C) critical for Hcp1 secretion in *P. aeruginosa* [29]. Thus, we generated various *fha* mutants, including the FHA-domain deletion mutant (*fha* ^Δ^ ^FHA^) and alanine substitution mutants at Arg 30 residue (*fha* ^R30A^), Ser 46 residue (*fha* ^S46A^), and both residues (*fha* ^R30AS46A^) to determine the roles of the FHA domain and its putative pThr binding residues in Hcp secretion. Hcp secretion was completely abolished in Fha^Δ^ ^FHA^ and Fha^R30AS46A^ strains, whereas Fha^R30A^ and Fha^S46A^ caused reduced Hcp secretion as compared to the wild-type C58 (Figure 1D). Because the protein abundance remained the same in all Fha point mutation variants as in the wild type (Figure 2A and Figure S1), we suggested that the pThr binding ability of Fha is indeed critical for Hcp secretion. Importantly, the evidence that the protein abundance of all analyzed T6SS components in these *fha* mutants remained at wild-type levels as well as in the absence of *ppkA* and *tagF-pppA* strongly suggests a PpkA- and Fha-dependent post-translational regulatory pathway for Hcp secretion from *A. tumefaciens* (Figure S1).

### TssL as a phosphoprotein that is phosphorylated at Thr 14 in a PpkA-dependent manner

To date, Fha-family protein is the only T6SS component identified to be phosphorylated by PpkA [28], [29]. In *P. aeruginosa*, Fha1 is phosphorylated by PpkA, and its Thr 362 phosphorylation site is required for Hcp secretion [29]. This PpkA-dependent Thr phosphorylation on Fha was also recently identified by a phosphoproteome screen and found to be critical for T6SS activation in *S. marcescens* [28], which suggests a common TPP in regulating type VI secretion. However, this Thr phosphorylation site is not universally conserved because no corresponding Thr could be identified in the *A. tumefaciens* Fha (Figure S2). Thus, PpkA may phosphorylate Fha at other sites or other T6SS component(s), in which its phosphorylation status is critical for interaction with Fha and activating type VI secretion in *A. tumefaciens.* Western blot analysis revealed that only TssL but not other analyzed T6SS components had slower migration in the wild-type C58 as compared with the Δ*ppkA* mutant (Figure 2A and Figure S1; marked with an asterisk), which suggests that TssL may be phosphorylated by PpkA.

To determine whether TssL is indeed phosphorylated, His-tagged TssL, which is functional in mediating the secretion of Hcp [20] and the putative T6SS toxin effector Atu4347 [6], [11] (Figure S3A), was expressed and purified by Ni-NTA resins for phosphorylation analysis. We first used Phos-tag SDS-PAGE for unambiguous separation of phosphorylated and unphosphorylated proteins on the gel. Western blot analysis revealed two TssL-His protein bands, with one faint lower band and one major upper band (Figure 2B). Importantly, with calf intestinal alkaline phosphatase (CIAP) treatment, the upper TssL-His band disappeared and the lower band intensity increased. Therefore, the upper protein band represented the phosphorylated TssL (*p*-TssL-His) and the lower band the unphosphorylated form (Figure 2B). Similar results were observed when total protein extracts were used for analysis (Figure S3B). Thus, TssL is indeed phosphorylated as the major form in *A. tumefaciens* when T6SS is activated in the acidic condition.

**Figure 3:**
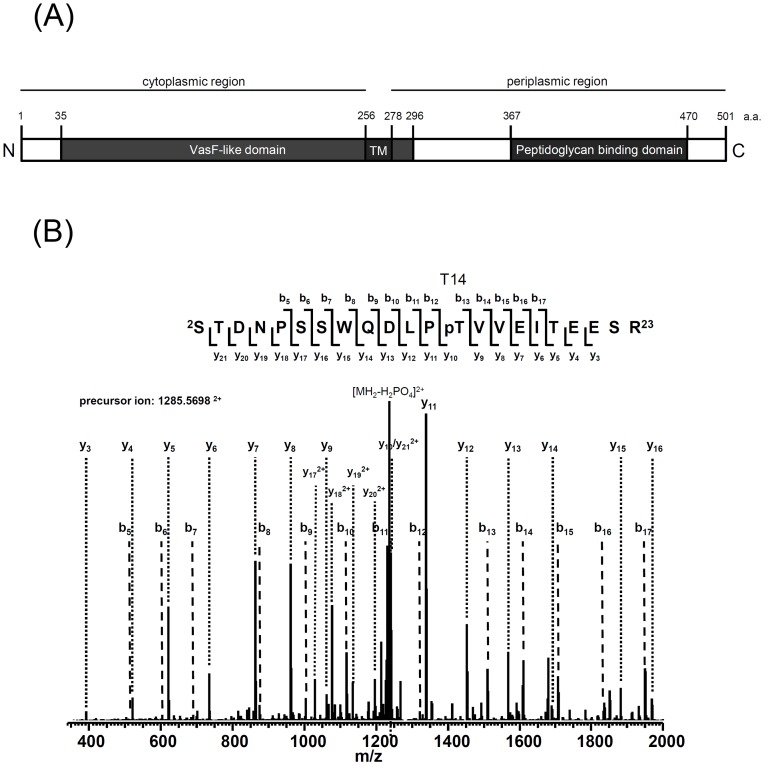
Mass spectrometry identification of TssL phosphorylation at Thr 14. (**A**) TssL domain organization presented according to the topology analysis by Ma et al. (2009) [20] and information from the NCBI database (http://www.ncbi.nlm.nih.gov/). TssL is an integral inner membrane protein (1–501 aa) with an N-terminal conserved IcmH/DotU VasF-like domain (35–296 aa) harboring one transmembrane domain (TM, 256–278 aa) and C-terminal peptidoglycan binding domain (367–470 aa) located in periplasm. (**B**) TssL-His purified from Δ*tssL*(pTssL-His) was separated by 12% SDS-PAGE, and Coomassie blue-stained TssL-His protein band was excised for in-gel digestion followed by mass spectrometry (MS) analysis. MS/MS ion spectrum with the matched b and y ions of the pT14-containing tryptic peptide STDNPSSWQDLPpTVVEITEESR in TssL-His is shown.

**Figure 4:**
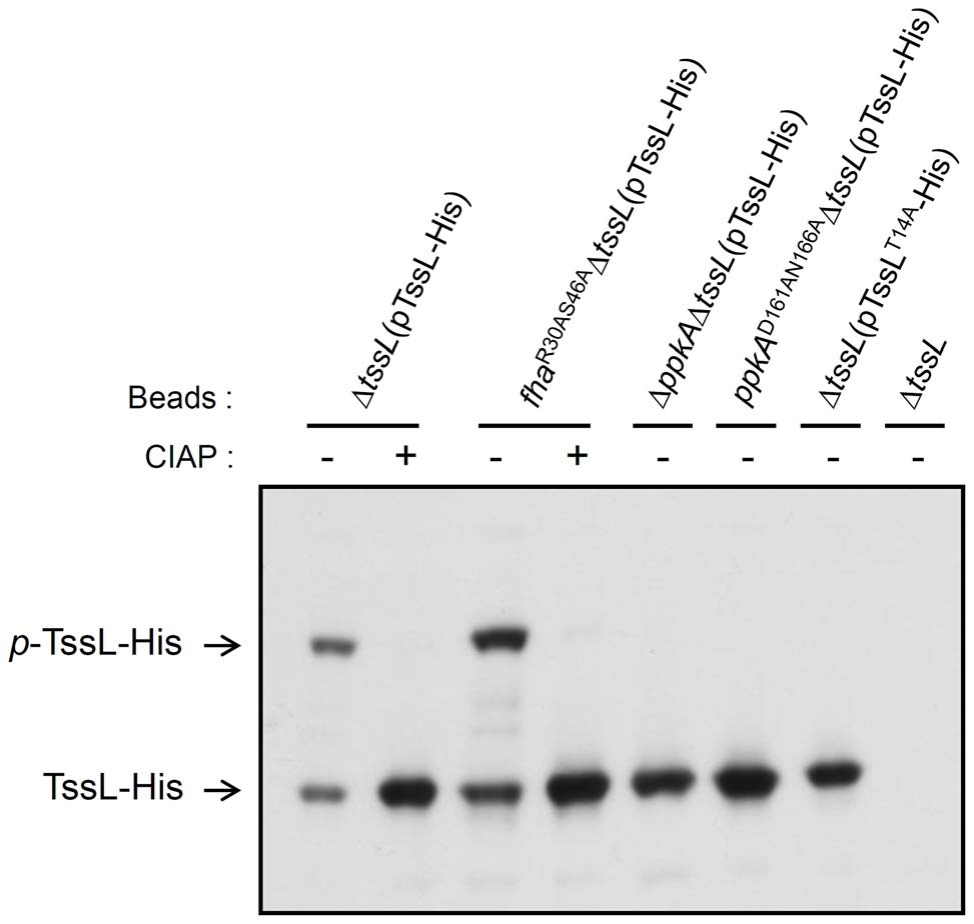
Phos-tag SDS-PAGE for TssL phosphorylation analysis in various mutants. Detection of the phosphorylation status of TssL-His on Phos-tag gel. Western blot analysis of the same volumes of Ni-NTA resins (40 µl) associated with TssL-His from different strains treated with (+) or without (−) CIAP and examined with specific antibody against 6×His. Total proteins isolated from Δ*tssL* were a negative control. Phos-tag SDS-PAGE revealed the upper band indicating the phosphorylated TssL-His (*p*-TssL-His) and lower band indicating unphosphorylated TssL-His.

In *A. tumefaciens*, TssL is a bitopic inner-membrane protein with an N-terminal VasF-like domin (35–296 aa) facing the cytoplasm and a C-terminal peptidoglycan binding domain (367–470 aa) exposed to the periplasm (Figure 3A) [20]. To identify the phosphorylated amino acid of TssL, the TssL-His expressed in *A. tumefaciens* was purified for phosphorylation site mapping by mass spectrometry (MS). MS analysis of purified TssL-His proteins identified phosphorylated Thr 14 (T14) located within its N-terminal cytoplasmic domain (residues 1–255) (Figure 3A, 3B) [20]. Because Thr 14 was the only phosphorylation site identified by MS/MS ion search with between 75% and 85% coverage of TssL-His proteins from 3 independent experiments (Figure S4), Thr 14 may be the sole phosphorylation site of TssL. This conclusion is also supported by the absence of a *p*-TssL-His protein band on Phos-tag gel when the TssL Thr 14 residue was substituted with alanine (A) (TssL^T14A^) (Figure 4). Therefore, TssL is a phosphoprotein phosphorylated at Thr 14.

To investigate whether PpkA kinase activity is required for TssL phosphorylation, we next generated a *ppkA* mutant encoding PpkA^D161AN166A^ with alanine substitutions in 2 conserved Asp 161 (D161) and Asn 166 (N166) residues located within the kinase magnesium binding loop (Figure S5) [42]. The corresponding Asp 129 of PpkA was previously demonstrated to be critical for Fha1 phosphorylation in *P. aeruginosa* [30]. In support of the role of PpkA and its kinase activity in phosphorylating TssL, we detected only an unphosphorylated TssL-His protein band in Δ*ppkA*Δ*tssL* and *ppkA* ^D161AN166A^Δ*tssL* expressing TssL-His, as shown by Phos-tag SDS-PAGE (Figure 4). Moreover, no phosphorylated residues could be identified by MS analysis of TssL-His proteins purified from *ΔppkAΔtssL* and *ppkA* ^D161AN166A^Δ*tssL* (data not shown). Not surprisingly, purified TssL-His remained with the wild-type phosphorylation pattern in *fha* ^R30AS46A^ by Phos-tag gel (Figure 4) and MS analysis (data not shown), and cellular TssL expressed in various *fha* mutants migrated to the same position as that for C58 on regular SDS-PAGE (Figure 2A and Figure S1). In conclusion, we provide evidence that PpkA kinase activity is required for TssL phosphorylation.

### PpkA kinase activity and phosphorylation of TssL at Thr 14 are critical for type VI secretion

**Figure 5:**
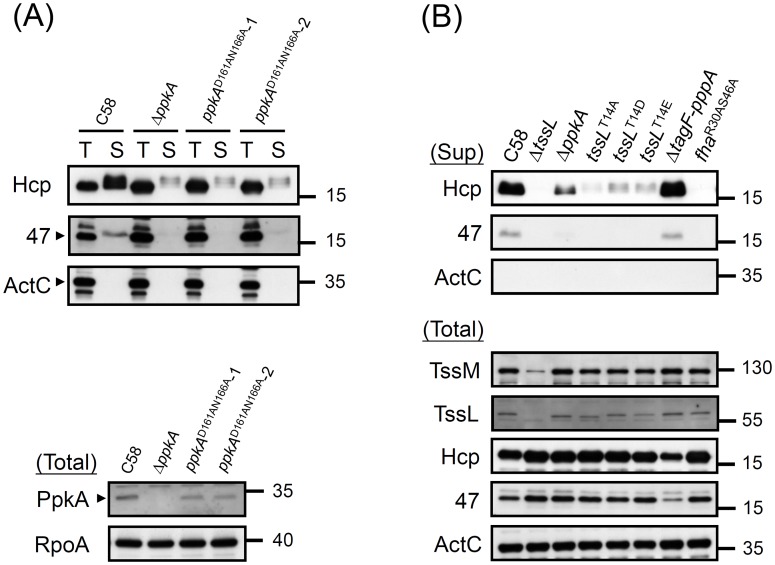
Hcp and Atu4347 secretion assays of various PpkA and TssL phosphorylation site mutations. (**A**) Hcp and Atu4347 secretion assay in *ppkA* mutants. The wild-type C58, Δ*ppkA*, and *ppkA* with D161AN166A substitutions strains (2 independent alleles) were analyzed for type VI secretion. (**B**) Type VI secretion and western blot analyses in various *tssL* mutants. Western blot analysis of total and secreted (Sup) proteins isolated from various *A. tumefaciens* strains with specific antibodies. ActC and RpoA were internal controls.

The requirement of PpkA and its conserved kinase catalytic motif for TssL phosphorylation strongly suggests the importance of TssL phosphorylation in regulating type VI secretion in *A. tumefaciens*. Thus, we determined whether the disruption of PpkA kinase catalytic sites affects type VI secretion activity, which was monitored by the secretion of Hcp [20] and the putative T6SS toxin effector Atu4347 [6], [11]. PpkA^D161AN166A^, which is incapable of phosphorylating TssL (Figure 4), also caused the largely reduced Hcp and Atu4347 secretion levels similar to that in Δ*ppkA* (Figure 5A). Although PpkA^D161AN166A^ protein levels are slightly lower than in wild-type C58, the complete loss of phosphorylated TssL in this kinase mutant strongly suggests that PpkA kinase activity plays a crucial role in regulating type VI secretion.

Next, we investigated whether TssL phosphorylation status is critical in regulating type VI secretion. In addition to generating the phosphorylation-inactive mutant TssL^T14A^ (Figure 4), we generated 2 additional *tssL* mutants with aspartic acid (D) or glutamic acid (E) substitutions (TssL^T14D^ and TssL^T14E^). We observed complete loss of Hcp secretion from Δ*tssL* and *fha* ^R30AS46A^ and low levels of secreted Hcp proteins from Δ*ppkA* and TssL phosphorylation site mutants (TssL ^T14A^, TssL^T14D^, and TssL^T14E^) (Figure 5B). Consistent with reduced Hcp secretion in Δ*ppkA* or TssL phosphorylation site mutants, Atu4347 secretion was highly attenuated and barely detected in the same mutants. Therefore, we suggested that TssL phosphorylation status is critical in regulating type VI secretion. The complete loss of both Hcp and Atu4347 secretion in *fha* ^R30AS46A^ suggests an essential role of the Fha pThr binding site in type VI secretion.

### Fha specifically binds Thr 14-phosphorylated TssL *in vitro*

**Figure 6:**
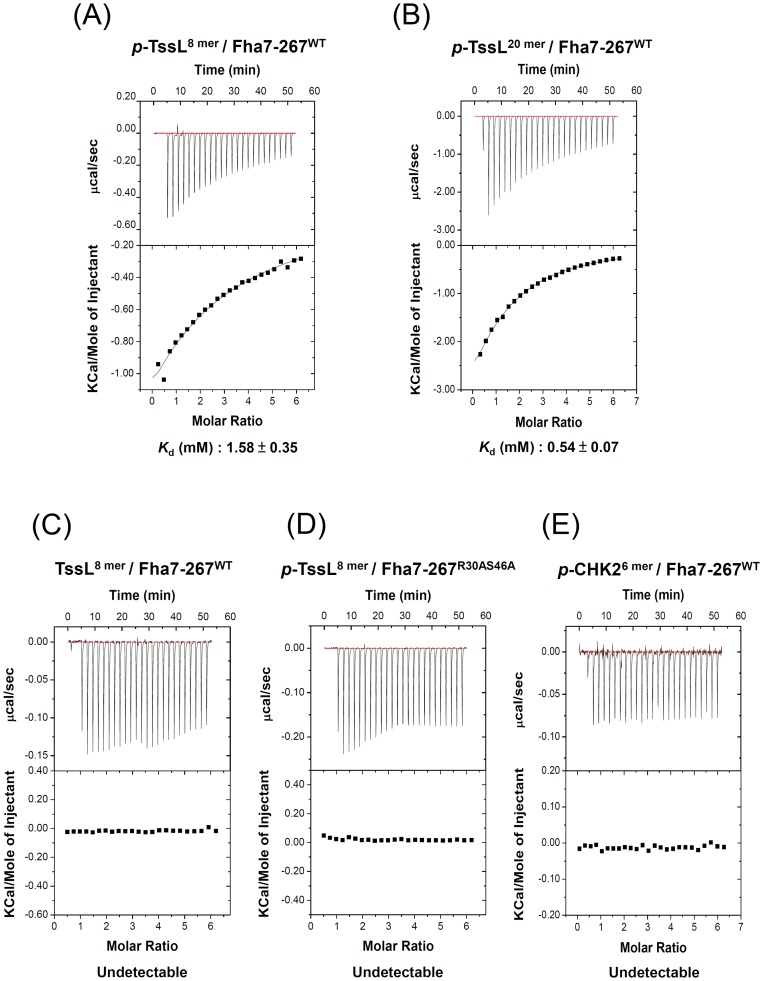
Phosphorylated TssL is a direct binding partner of Fha. Isothermal titration calorimetry (ITC) of specific binding between Fha and TssL peptides. Purified Fha7-267^WT^ (**A, B, C, E**) or Fha7-267^R30AS46A^ (**D**) was titrated with synthetic phosphorylated TssL (DLPpTVVEI, *p*-TssL^8 mer^ or DNPSSWQDLPpTVVEITEESR, *p*-TssL^20 mer^), unphosphorylated TssL (DLPTVVEI, TssL^8 mer^), or phosphorylated CHK2 (VSpTQEL, *p*-CHK2^6 mer^) [43] peptides. The equilibrium dissociation constant (*K_d_*) between truncated Fha7-267 proteins and synthetic peptides is shown at the bottom. Other combinations without any detectable binding ability are shown as undetectable. Top panel shows the raw calorimetric data for the interaction and bottom panel the integrated heat changes, corrected for heat of dilution, and fitted to a single-site binding model.

Because FHA domains are well known to bind to pThr residues specifically [33]–[35], we hypothesized that Fha specifically binds Thr 14-phosphorylated TssL to activate type VI secretion. To test this hypothesis, we first performed isothermal titration calorimetry (ITC) analysis with purified Fha proteins and synthetic phosphorylated- and unphosphorylated TssL N-terminal peptides. Because of the instability of full-length Fha protein purified from *E. coli* (data not shown), we purified truncated Fha proteins with different lengths for the ITC experiments (Figure S6A). Fha7-267^WT^ containing the intact FHA domain was capable of binding to the 8 mer phosphorylated TssL peptide (DLPpTVVEI, *p*-TssL^8 mer^), although with relatively low affinity (*K_d_* 1.58±0.35 mM) (Figure 6A). The binding affinity was ∼3-fold higher (*K_d_* 0.54±0.07 mM) with a longer phosphorylated TssL peptide (DNPSSWQDLPpTVVEITEESR, *p*-TssL^20 mer^) (Figure 6B). These data suggest that the TssL sequence or structure adjacent to the phosphorylation peptide motif contributes to formation of the *p*TssL-Fha complex. We could not detect the binding affinity with the unphosphorylated TssL peptide (DLPTVVEI, TssL^8 mer^) or the 6 mer phosphorylated CHK2 peptide (VSpTQEL, *p*-CHK2^6 mer^) as controls [43] (Figure 6C, 6E). Notably, the truncated Fha proteins with a mutated FHA domain (Fha7-267^R30AS46A^) completely lost the ability to interact with the 8 mer *p-*TssL peptide (Figure 6D). We showed specific binding to the *p-*TssL peptide but not unphosphorylated TssL peptide with a longer version of the truncated Fha protein (Fha7-309^WT^) (Figure S6B). These data strongly suggest that Fha specifically binds to Thr 14-phosphorylation TssL peptide via its pThr binding motif of the FHA domain. The increased binding affinity for the FHA domain with a longer phosphorylated TssL peptide also suggests that stronger interaction may occur *in vivo*, where Fha interacts with full-length TssL. To our knowledge, this is the first demonstration of specific binding of an FHA domain-containing protein to the T6SS phosphorylated target.

### Fha specifically interacts with phosphorylated TssL, and this complex is critical for efficient interactions of TssL with Hcp and Atu4347

**Figure 7:**
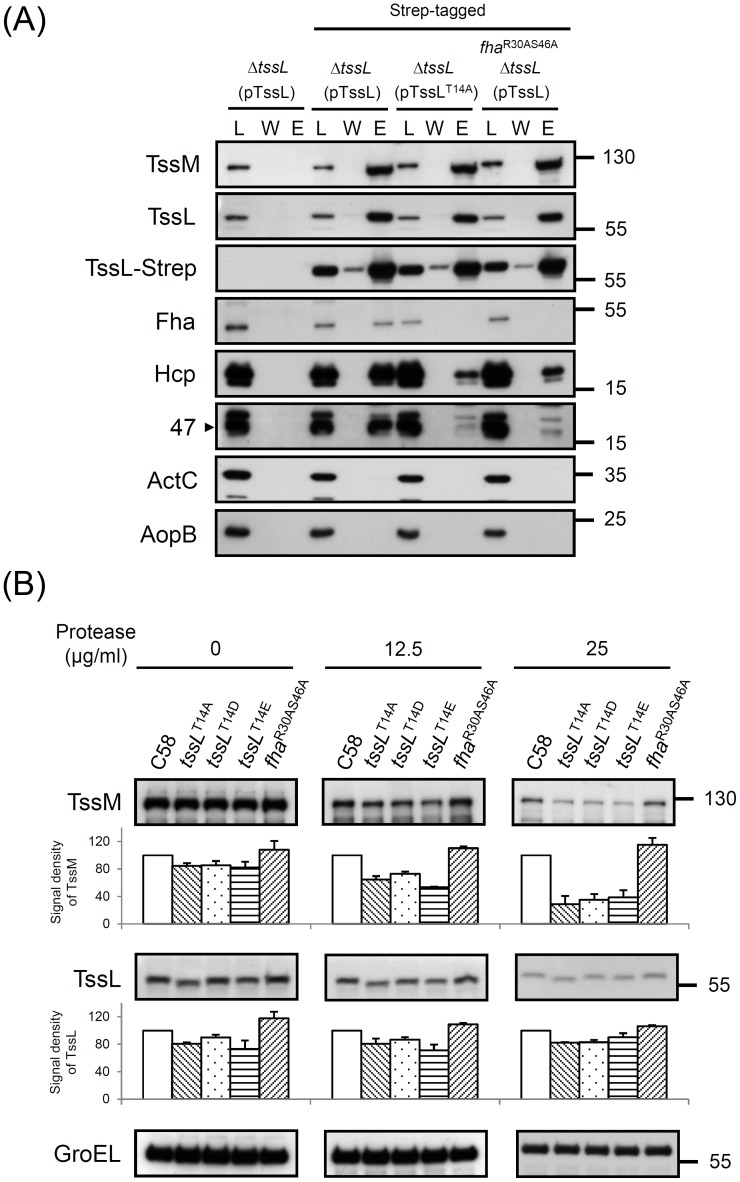
TssL-Strep pulldown and spheroplast protease susceptibility assays in *A. tumefaciens*. (**A**) Pulldown assay by TssL-Strep in various *A. tumefaciens* strains. Western blot analysis of the loaded Triton X-100 solublized protein fraction (L), wash (W), and elution (E) examined with specific antibodies. The soluble protein ActC and outer-membrane protein AopB [46] were internal controls. (**B**) Spheroplasts from various *A. tumefaciens* strains were incubated with different protease concentrations as indicated. The degree of susceptibility or resistance to protease in various strains was analyzed by western blot analysis with specific antibodies. Cytoplasmic GroEL [47] was an internal control for resistance to protease digestion. The amount of analyzed proteins was further quantified by use of UVP BioSpectrum 600 and normalized to the internal control GroEL. The relative intensity from TssM and TssL are shown at the bottom of analyzed strains by setting the wild-type C58 level to 100. The quantitative results shown with standard deviation were obtained and normalized from at least 2 independent experiments.

Next, we investigated the *p-*TssL-Fha complex formation *in vivo* by pulldown assays using functional Strep-tagged TssL (Figure S3A). To avoid the non-specific protein–protein interactions occurring when proteins are released into solution after cell breakage and to detect weak and dynamic interactions, we used the cleavable and membrane permeable cross-linker dimethyl 3,3′-dithiobispropionimidate (DTBP) to cross-link interacting proteins before cell lysis [44] for interaction studies in *A. tumefaciens.* The known TssL-interacting inner-membrane protein TssM [20], [22] co-precipitated with TssL-Strep in all analyzed samples (Figure 7A), which indicates that TssL interacts with TssM independent of its phosphorylation status or association with Fha. In contrast, Fha was pulled down by wild-type TssL-Strep but not TssL-Strep with a T14A substitution or by TssL-Strep in the Fha^R30AS46A^ strain. All interaction proteins could not be co-precipitated by Strep-Tactin resin from the negative control, Δ*tssL* expressing wild-type TssL without Strep-tag. As well, the two non-T6SS proteins, soluble protein ActC [20], [45] and outer membrane protein AopB [46], were not pulled down with TssL-Strep (Figure 7A).

Our previous study showed that Hcp is recruited into the TssM-TssL complex via directly interacting with the TssL [22]. Thus, we determined whether TssL phosphorylation status and its specific binding to Fha are critical for its interaction with Hcp. Abundant Hcp was pulled down by wild-type TssL-Strep but only small amounts of Hcp bound to TssL^T14A^-Strep or wild-type TssL-Strep in the Fha^R30AS46A^ strain (Figure 7A). Because TssL remained highly phosphorylated in the Fha^R30AS46A^ strain (Figure 4), these data suggest that Fha binding to *p-*TssL is critical for efficient Hcp-TssL interactions. Interestingly, Atu4347 was also co-purified with TssL, in which its association with TssL was stimulated by phosphorylation of TssL and pThr binding ability of Fha (Figure 7A).

### TssL phosphorylation is required to trigger a conformational switch of TssM ATPase

Because TssM undergoes an ATP binding-induced conformational change and the subsequent ATP hydrolysis energizes Hcp recruitment into the TssM-TssL complex and activates Hcp secretion [22], we next tested whether TssL phosphorylation and/or its interaction with Fha is required for TssM structure transition. TssM undergoes a structural transition on ATP binding, as seen by the higher protease resistance of wild-type TssM than the ATP binding-deficient walker A mutant [22]. Thus, we performed a spheroplast protease susceptibility assay to determine whether the TssM becomes more susceptible to protease digestion in mutants expressing unphosphorylated TssL or *fha* ^R30AS46A^. The relative protein abundance of TssL and TssM was quantified and normalized with an internal control, the cytoplasmic protein GroEL resistant to protease digestion [22], [47]. Protein abundance of both TssL and TssM was slightly lower in all three TssL phosphorylation site mutants (TssL^T14A^, TssL^T14D^, and TssL^T14E^) as compared with the wild type and *fha* ^R30AS46A^ strains before protease treatments (Figure 7B). However, no further increased degradation could be detected for 3 TssL variants with phosphorylation-site mutations relative to that of wild-type TssL. In contrast, TssM protein abundance was further dose-dependently decreased in the TssL phosphorylation-site mutants after protease treatment (Figure 7B). Differently, TssM remained with the same degree of protease resistance in *fha* ^R30AS46A^ regardless of protease concentration used. These data suggest that TssL phosphorylation but not *p-*TssL-Fha complex formation is responsible for TssM conformational change.

## Discussion

Our discovery of TssL as a new PpkA substrate in *A. tumefaciens*, together with previous findings of PpkA-mediated Fha phosphorylation from *P. aeruginosa* [29], [30] and *S. marcescens* [28], have revealed both the conservation and uniqueness of Thr phosphorylation regulatory pathways in different bacteria. These studies demonstrate that both PpkA kinase activity and phosphorylation of target proteins are critical for type VI secretion. In addition, the pThr binding motif of Fha proteins from both *P. aeruginosa* and *A. tumefaciens* are crucial for Hcp secretion, which indicates the importance of this Thr phosphorylation event and that Fha interaction with phosphoprotein is a conserved and key step for subsequent type VI assembly and secretion. However, although the Fha Thr phosphorylation site is conserved in several Fha-family proteins across different bacterial species [28], we could not detect the corresponding Thr in the *A. tumefaciens* Fha protein sequence (Figure S2). Our attempt to detect Fha phosphorylation by Phos-tag SDS-PAGE was also unsuccessful (Figure S3C), which suggests that Fha may not be the phosphorylated target of PpkA in *A. tumefaciens*. The N-terminal cytoplasmic domain harboring the Thr 14 (T14) phosphorylation site of TssL is highly conserved in all analyzed *Agrobacterium* and *Rhizobium* species that also encode Thr phosphorylation components (Figure S7) but not in other TssL orthologs encoded by distantly related bacterial species (Figure S8). However, Thr residues are widely distributed at the N-terminal domain of all analyzed TssL-family proteins across different bacterial species (Figure S8). In considering the sequence diversity of the FHA domain recognition site (pT+3) [34], [35], TssL may be a conserved phosphorylation target of PpkA not only in *Agrobacterium* and *Rhizobium* species but also in other bacteria outside of Rhizobiaceae, which suggests a broader function of TssL phosphorylation in regulating type VI activity.

Low but detectable Hcp secretion from the absence of PpkA or PpkA with mutation in its catalytic site suggested that PpkA kinase activity is not absolutely required to activate type VI secretion in *A. tumefaciens*. This finding is consistent with the low Hcp1 secretion when *ppkA* was deleted in the *P. aeruginosa* wild-type strain, in which a Thr phosphorylation-independent pathway could be derepressed by the deletion of *tagF* [27], [31]. The presence of a TagF-PppA fusion protein encoded by the T6SS gene cluster suggested that T6SS may be negatively regulated by TagF in a Thr phosphorylation-independent manner in *A. tumefaciens*. In *P. aeruginosa*, Thr phosphorylation is stimulated by a cue during the surface growth [27], which implies a signal derived from the cell-to-cell contact. Indeed, the Thr phosphorylation regulatory components PpkA, PppA, and TagT are required for the activation of the *P. aeruginosa* T6SS dueling activity when encountering the T6SS attack by contacted *V. cholerae* cells [17]. The authors suggested that Thr phosphorylation-positive regulation by TagQRST localized in the membranes [30], [31] may function to perceive and transduce signals to PpkA for activation of the local T6SS assembly for counterattack [17]. In *A. tumefaciens*, Thr phosphorylation is not absolutely required but plays an important role to enhance type VI secretion activity. Less than 5% of Fha1 is phosphorylated in *P. aeruginosa* grown in liquid culture and up to ∼20% of *p*-Fha1 is stimulated by growth on solid-phase medium [27]; however, most if not all of TssL is phosphorylated when *A. tumefaciens* is grown in acidic liquid culture. Moreover, the absence of *ppkA* and loss of TssL Thr phosphorylation caused a similar degree of reduced Hcp and Atu4347 secretion when *A. tumefaciens* was grown on agar (Figure S9) and liquid culture (Figure 5B). Therefore, surface-derived cell contact may not be a signal to stimulate the Thr phosphorylation event in *A. tumefaciens.* This notion is also supported by the lack of TagQRST orthologs in *A. tumefaciens*. From the presence of both Thr phosphorylation-independent and -dependent type VI activation in *A. tumefaciens*, we propose that the initial acid-induced type VI gene expression may somehow trigger a signal to activate TPP. The signal may come from the assembly of the T6SS subassembly from the same cell or by sensing the secretion activity from other cells. However, the evidence that TssL remains highly phosphorylated even when type VI secretion is abolished with the loss of Fha or its functional FHA domain suggests that the signal is unlikely to be Hcp or effectors secreted from neighboring cells. Alternatively, the signal may be derived from the acid-induced but T6SS-independent components [48]–[50]. Future work to identify the genetic requirements stimulating Thr phosphorylation will shed light on the molecular mechanisms and biological significance of this posttranslational regulation in *A. tumefaciens*.

Only the authentic phosphate group at Thr 14 but not the phospho-mimic residues exhibiting the full function of TssL in activating type VI secretion is consistent with the notion that the FHA-domain phosphopeptide binding site is highly pThr-specific [51], [52]. Interestingly, Hcp secretion was slightly higher from Δ*ppkA* than from all 3 TssL phosphorylation site mutants, with almost no detection of Hcp secretion in the T14A mutant (Figures 5B and S9), which suggests that these Thr 14 (T14) substitutions may partially affect other TssL function(s) besides its phosphorylation status. The observation that all 3 TssL variants are less stable and subsequently affect the stability of TssM (Figure 7B) suggests that either Thr 14 or its phosphorylation status affects TssL stability. Because TssM is highly unstable in the absence of TssL [20], [22] but remains at similar levels in Δ*ppkA* as in the wild type grown in liquid culture or on agar (Figure 5B and S9), the reduced stability of TssL and TssM may be due to the substitution of T14 instead of TssL phosphorylation status. However, the slightly reduced TssL and TssM protein levels in the TssL phosphorylation site mutants cannot account for the high attenuation of Hcp and Atu4347 secretion because overexpression of TssL^T14A^ was unable to restore Hcp or Atu4347 secretion in Δ*tssL* as did the wild type TssL (Figure S10). Furthermore, while the 3 TssL variants remain the same protease resistance level like wild-type TssL, TssM become more susceptible to protease in these TssL phosphorylation-site mutants. These data suggested that TssL phosphorylation status indeed affect TssM conformational change. Thus, we conclude that PpkA kinase activity and phosphorylation of TssL at Thr 14 are critical for TssM conformational change and type VI secretion.

Why Hcp and Atu4347 secretion is completely abolished in the *fha* ^R30AS46A^ mutant but the loss of TssL phosphorylation in Δ*ppkA* retains low but detectable Hcp secretion activity is unclear (Figure 5B and S9). Because no additional Ser/Thr kinase gene could be identified in the *A. tumefaciens* C58 genome by annotation and BLAST analysis (data not shown) and no phosphorylation of TssL could be detected in Δ*ppkA* (Figure 4), PpkA may be the sole Thr kinase in *A. tumefaciens.* Therefore, the complete loss of type VI secretion activity in the *fha* ^R30AS46A^ mutant is unlikely due to the loss of its ability in binding to another pThr site other than TssL.

The formation of the TssM-TssL inner-membrane complex independent of TssL phosphorylation is consistent with the detection of TssM-TssL interaction when expressed in heterologous *E. coli* [20], [22]. Although we could not detect a conformation change for TssL by protease susceptibility assay, TssL may undergo a structural transition on phosphorylation by PpkA, thereby leading to the accessibility or increased affinity of interacting TssM for ATP binding and trigger the structural transition of TssM. The detection of the ATP binding-induced TssM conformational change in the Fha^R30AS46A^ strain suggests that Fha binds to the *p-*TssL after TssM binds with ATP. Furthermore, Hcp and Atu4347 can only bind efficiently with TssL when TssL is phosphorylated and forms the *p-*TssL-Fha complex (Figure 7A). Together with previous findings that TssM and its ATPase activity are required for recruiting Hcp into TssM-TssL complex via direct interaction with TssL and activating Hcp secretion [22], we suggested that Fha likely interacts with *p-*TssL residing in *p-*TssL-TssM inner membrane complex instead of *p-*TssL alone. To investigate whether TssM is required to trigger TssL phosphorylation by PpkA and subsequently causes the reduced Hcp and Atu4347 binding to TssL, we determined the phosphorylation status of TssL in the *tssM* deletion mutant by regular and Phos-tag SDS-PAGE. To our surprise, TssL remains phosphorylated at wild-type levels in the absence of TssM in *A. tumefaciens* (Figure S11). These data indicated that TssM and its ATPase activity are not required for TssL phosphorylation but critical for recruiting Hcp and Atu4347 into TssM-*p-*TssL-Fha ternary complex for type VI assembly and secretion.

**Figure 8:**
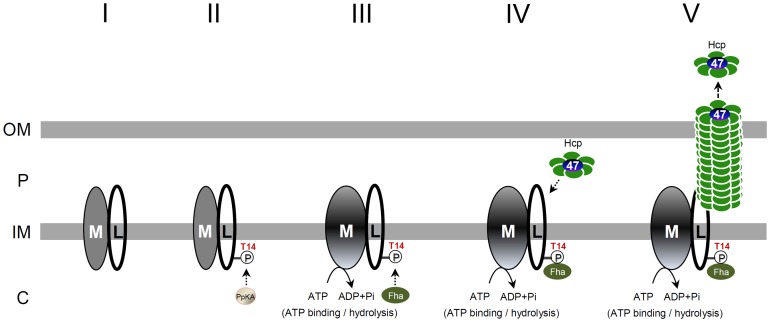
A proposed model of TssL phosphorylation-induced type VI assembly and secretion pathway. The regulatory switch is performed by PpkA (Ser/Thr kinase), Fha (FHA domain-containing protein), TssL (L, bitopic inner-membrane protein), and TssM (M, inner-membrane ATPase). The TssM-TssL inner-membrane complex likely forms (step I) before TssL phosphorylation by PpkA. PpkA phosphorylates the Thr 14 residue on TssL (step II) and triggers the ATP binding-induced conformational change of TssM (step III), followed by direct binding to the FHA domain of Fha at the T14 phosphorylation site (step IV). The formation of the TssM-*p-*TssL-Fha complex in conjunction of TssM ATP hydrolysis may stimulate the recruitment of Hcp and Atu4347 into the complex and activates Hcp-Atu4347 secretion across the outer membrane to the extracellular milieu (step V). From our previous Hcp-Atu4347 interaction studies and recent evidence of Hcp as an effector receptor [11], [12], Hcp may form a hexamer receptor binding Atu4347 in the inner ring before interacting with TssM-*p-*TssL-Fha subassembly. The evidence of direct interaction of Hcp with the periplasmic domain of TssL [22] also implied that TssM-*p-*TssL-Fha subassembly interacts with Hcp and Atu4347 in periplasm but further detailed studies are required. OM, outer membrane; IM, inner membrane; P, periplasm; C, cytosol.

Taken together, we proposed a hypothetical model for the TssL phosphorylation-induced T6SS assembly pathway (Figure 8). The formation of the TssM-TssL inner-membrane complex likely occurs (step I) before phosphorylation of TssL (step II). TssL phosphorylation triggers TssM conformation switch (step III), which occurs before cytoplasmic Fha is recruited into the TssM-*p-*TssL complex via interacting with N-terminal Thr 14 phosphopeptide motif of TssL (step IV). The formation of this TssM-*p-*TssL-Fha complex combined with TssM ATP hydrolysis activates the efficient recruitment of Hcp and Atu4347 into this T6SS inner-membrane subassembly for type VI tube assembly and effector secretion across outer membrane (step V). The direct evidence for formation of this inner-membrane subassembly remains lacking and other alternative or multiple pathways may exist. Future work to investigate whether and how TssM ATP binding/hydrolysis influence Fha recruitment to activate type VI complex formation and secretion shall provide further mechanistic insights in type VI assembly and secretion.

Recent phosphoproteomic analysis in *S. marcescens* identified Fha but not TssL as PpkA-phosphorylated substrate [28]. Thus, the phosphorylation events for Fha and TssL have not been concurrently identified in the same organism. However, we do not exclude that both proteins may be phosphorylated and coordinated to regulate type VI assembly and effector secretion. In *P. aeruginosa*, Fha1 focally co-localizes with ClpV1 and is required for ClpV1 focal recruitment [29] and dynamics [17], which suggests that Fha1 is a T6SS core scaffold protein. The requirement of *tssM* (*icmF*) for ClpV1 foci recruitment in *P. aeruginosa* [53] indeed supports our proposed model for the recruitment of Fha into TssM-*p-*TssL inner-membrane complex. Our findings now extend the knowledge that phosphorylation of TssL may function to initiate the inner-membrane subassembly formation, for which Fha may come into play at a later stage for recruiting ClpV1 for TssB-TssC tubule disassembly. Future work to determine the conservation or uniqueness of Fha and TssL phosphorylation in different bacteria and to investigate their hierarchy and roles for type VI complex assembly will elucidate the mechanistic role of Thr phosphorylation in T6SS. Only a subset of T6SS-containing bacterial species encodes the Thr phosphorylation components [1], [26], [27], so Thr phosphorylation-dependent regulation may be required for only certain bacteria that encounter complex environmental signals. The evolution of the conservation and divergence of Thr phosphorylation components, phosphorylated targets, and regulatory mechanisms in different bacterial species is an interesting question for future study.

## Materials and Methods

### Bacterial strains, plasmids, and growth conditions

Strains, plasmids, and primer sequences used in this study are in Tables S1 and S2. *A. tumefaciens* and *Escherichia coli* strains were grown at 28°C in 523 [54] and at 37°C in LB [55], respectively. The plasmids were maintained by the addition of 50 µg/ml gentamycin (Gm) for *A. tumefaciens* and 100 µg/ml ampicillin (Ap), and 50 µg/ml Gm for *E. coli.*

### Hcp and Atu4347 secretion assay

*A. tumefaciens* cells grown in liquid AB-MES medium (pH 5.5) [56] at 25°C for 6 hr were concentrated by trichloroacetic acid precipitation (TCA) for Hcp and Atu4347 secretion assays as described [20], [41].

### Plasmid construction and generation of in-frame deletion or amino acid substitution(s) mutants

All in-frame deletion and amino acid substitution(s) mutants were generated in *A. tumefaciens* C58 via double crossover using the suicide plasmid pJQ200KS [57] as described [20], [41]. The detailed procedures for the construction of plasmids and mutant strains are described in Supporting Information S1.

### Dephosphorylation analysis

Dephosphorylation analysis by calf intestinal alkaline phosphatase (CIAP) was performed according to the user manual (New England Biolabs, Beverly, MA, USA) with minor modifications. Equal amounts of Ni-NTA resins with purified TssL-His or total protein extracts isolated from various *A. tumefaciens* strains were resuspended in 1× CIAP buffer containing 100 mM NaCl, 50 mM Tris-HCl (pH 7.9), 10 mM MgCl2, 1 mM DTT, and 1× protease inhibitor cocktail (EDTA-free) with CIAP at 1 unit per µg of protein. The protein samples treated with or without CIAP were incubated at 37°C for 90 min. An equal volume of 2× SDS loading buffer was added and incubated at 96°C for 20 min and analyzed by Phos-tag SDS-PAGE.

### Phos-tag SDS-PAGE analysis

The Phos-tag SDS-PAGE analysis was performed according to the user manual for Phos-tag Acrylamide AAL-107 (Wako Pure Chemical Industries, Osaka, Japan) with minor modifications. Protein samples were separated on 7% polyacrylamide gels containing 0.35 M Bis-Tris-HCl (pH 6.8), 35 µM Phos-tag Acrylamide AAL-107, and 100 µM ZnCl_2_, with electrophoresis conducted at 40 mA/gel under a maximum voltage of 90V in a cold room. After electrophoresis, Phos-tag gels were washed with transfer buffer (25 mM Tris, 192 mM glycine, 20% methanol) containing 1 mM EDTA for 15 min with gentle shaking followed by a second wash in transfer buffer without EDTA for 15 min. The gels were washed with transfer buffer containing 1% SDS for 15 min before transfer to PVDF membranes with a submarine blotting apparatus.

### In-gel digestion and mass spectrometry analysis

Phosphorylation site mapping was determined by in-gel trypsin or trypsin/chymotrypsin digestion of purified TssL-His followed by nanoLC/nanospray/tandem mass spectrometry (LC-ESI/MS/MS) analysis. TssL-His proteins expressed in *A. tumefaciens* were purified by use of Ni-NTA resins and separated by 12% SDS-PAGE followed by Coomassie blue staining. TssL-His protein bands were cut out for in-gel trypsin or trypsin/chymotrypsin digestion as described [58], [59]. The extracted tryptic peptides were subjected to the LC separation followed by a linear quadrupole ion trap-Fourier transform (LTQ-FT) ion cyclotron resonance mass spectrometer (Thermo Fisher Scientific) equipped with a nanoelectrospray ion source (New Objective, Woburn, MA, USA) for protein identification and phosphorylation site mapping.

### TssL-Strep pulldown assay in *A. tumefaciens*

To avoid the non-specific protein–protein interactions occurring when proteins are released into solution after cell breakage, we used the cleavable and membrane permeable cross-linker dimethyl 3,3′-dithiobispropionimidate (DTBP) to cross-link interacting proteins before cell lysis [44] for the TssL-Strep pulldown assays from *A. tumefaciens.* The pulldown assay with Strep-Tag was performed according to the user manual for StrepTactin Sepharose (GE Healthcare). In total, 500 ml *A. tumefaciens* cell cultures were centrifuged and washed 3 times with 12 ml phosphate buffer (20 mM sodium phosphate, pH 7.6; 20 mM sodium chloride) and resuspended in the same buffer adjusted to OD_600_ about 4. Membrane-permeable cross-linker dimethyl 3,3′-dithiobispropionimidate (DTBP) was added at a final concentration of 5 mM, and the mixture was incubated at room temperature for 45 min. The reaction was stopped by adding Tris-HCl (pH 7.6) to a final concentration of 20 mM for 15 min. The cells were collected by centrifugation and washed twice with 12 ml of 50 mM Tris-HCl (pH 7.6) before pulldown assay. The harvested cells were resuspended in binding buffer (100 mM Tris-HCl, pH 8.0; 150 mM NaCl; 1 mM EDTA) containing 0.05% Triton X-100 and underwent sonication on ice. The cell lysate was centrifuged twice at 10,000× g for 15 min at 4°C. The soluble fraction was passed through *Strep-*Tactin resins (GE Healthcare) and washed 6 times with binding buffer (100 mM Tris-HCl, pH 8.0; 150 mM NaCl; 1 mM EDTA) containing 0.05% Triton X-100. The bound proteins were eluted by use of elution buffer (100 mM Tris-HCl, pH 8.0; 150 mM NaCl; 1 mM EDTA; 2.5 mM desthiobiotin). The fractions were examined by western blot analysis.

### Isothermal titration calorimetry (ITC)

ITC analysis was used to determine the binding affinity and stoichiometry between Fha and TssL T-14 peptide with use of the MicroCal iTC_200_ system (GE Healthcare) and analyzed with ORIGIN software. Truncated His-tagged Fha^7–267^ and Fha^7–309^ proteins were overexpressed and purified from *E. coli* BL21(DE3) as described [43]. The proteins and various synthetic peptides were dissolved in buffer containing 50 mM Tris-HCl (pH 8.0), 150 mM NaCl. Synthetic phosphorylated TssL (DLPpTVVEI, *p*-TssL^8 mer^; DNPSSWQDLPpTVVEITEESR, *p*-TssL^20 mer^), unphosphorylated TssL (DLPTVVEI, TssL^8 mer^), and phosphorylated CHK2 (VSpTQEL, *p*-CHK2^6 mer^) [43] peptides, were used to titrate the various Fha^7–267^ or Fha^7–309^ proteins at 25°C with a total 25 times of injections with 200 µM protein in the sample cell and 6 mM peptide in the injection syringe. The titration heat was calculated to eliminate the effect of heat generated from titrating the ligand into buffer. Thermal data were fitted to the One Set of Sites binding model with the N value fixed at 1 to yield the value of the equilibrium dissociation constant (*K_d_*).

### Spheroplast preparation and protease susceptibility assay

Spheroplast preparation and protease susceptibility assay were performed as described [22] with modifications. *A. tumefaciens* cells were grown in liquid AB-MES medium (pH 5.5) [56] at 25°C for 6 hr. Cells were harvested by centrifugation at 10,000× g for 15 min at 4°C and washed once with 50 mM Tris-HCl (pH 7.5). To prepare spheroplasts, cell pellets were resuspended gently in buffer containing 50 mM Tris-HCl (pH 7.5), 20% sucrose, 2 mM EDTA, 0.2 mM DTT, and 1 mg/ml lysozyme and incubated on ice for 1 hr. After lysozyme treatment, spheroplasts were treated with *Streptomyces griseus* protease (Sigma) at a final concentration of 12.5 or 25 µg/ml for 10 min on ice in the presence of 10 mM MgSO_4_. The reaction was stopped by adding an equal amount of 2× SDS loading buffer and incubated at 96°C for 20 min before SDS-PAGE.

### Western blot analysis

Western blot analysis was performed as described [56] with primary polyclonal antibodies against proteins (PpkA, PppA, TssK, Fha, TssE, TssC_41_, TssB, TssA, ClpV, Atu4347, VgrGs, RpoA, AopB) [11], TssL [20], TssM [20], Hcp [41], ActC [45], GroEL [47], polyclonal antibodies against His (Sigma), or monoclonal antibodies against Strep (IBA-Life Sciences, Goettingen, Germany), followed by a secondary antibody horseradish peroxidase (HRP)-conjugated goat anti-rabbit IgG (chemichem) and detected by use of the Western Lightning System (Perkin Elmer, Boston, MA). Chemiluminescent bands were visualized on X-ray film (Kodak, Rochester, NY) or were quantified with the UVP BioSpectrum 600 imaging system (Level Biotechnology, Inc.).

## Supporting Information

Figure S1 **Western blot analysis of T6SS components in various strains.** Western blot analysis of total proteins from various *Agrobacterium tumefaciens* strains resolved by 10% or 12% Glycine-SDS-PAGE and examined with specific antibodies. The soluble protein ActC and RNA polymerase α subunit (RpoA) were internal controls. The proteins analyzed are indicated on the left, the molecular weight standards are on the right, and with arrows when necessary. The TssL protein band with faster migration in Δ*ppkA* mutant is marked with an asterisk.
(TIF)

Figure S2 **Alignment of the amino acid sequences of Fha orthologs.** Amino acid sequences of Fha-family proteins from *Pseudomonas aeruginosa* (Fha1/PA0081, GI: 15595279), *A. tumefaciens* (Fha/Atu4335, GI: 15890648), *Rhizobium leguminosarum* bv. *viciae* 3841 (FHA domain-containing protein, GI: 115253782), *R. leguminosarum* bv. *trifolii* WSM2297 (FHA domain-containing protein, GI: 393183378), *Rhodobacter sphaeroides* WS8N (FHA domain-containing protein, GI: 332561141), *Bradyrhizobium japonicum* (Blr3598, GI: 27351858), *Pseudomonas fluorescens* (Fha, GI: 68347667), *Shewanella frigidimarina* (FHA domain-containing protein, GI: 122299446), *Vibrio parahaemolyticus* (Hypothetical protein, GI: 28809344), *Xanthomonas axonopodis* pv. *citri* (Conserved hypothetical protein, GI: 21110543), and *Xanthomonas campestris* pv. *vesicatoria* (Conserved hypothetical protein, GI: 78036121). Identical amino acid residues are highlighted in black. Conserved amino acid residues in FHA domain used for mutagenesis are indicated with red asterisks, and Thr 362 phosphorylation site of Fha1 of *P. aeruginosa* is indicated with an arrowhead. Sequences were aligned and highlighted by use of ClustalW2 (http://www.ebi.ac.uk/Tools/msa/clustalw2/).
(TIF)

Figure S3 **Protein secretion assay and Phos-tag SDS-PAGE analysis.** (**A**) His-tagged or Strep-tagged TssL has full function in mediating Hcp and Atu4347 secretion. Western blot analysis of total (T) and secreted (S) protein isolated from various *A. tumefaciens* strains grown in AB-MES (pH 5.5) for 6 h at 25°C and separated by 12% Glycine-SDS-PAGE. The secreted proteins were collected from 1 ml of culture medium after removal of bacterial cells by centrifugation and were concentrated by TCA precipitation [41]. The non-secreted soluble protein ActC was an internal control. The proteins analyzed and molecular weight standards are on the left and right, respectively, and with arrows when necessary. (**B**) Phos-tag SDS-PAGE analysis of TssL-His. Total proteins isolated from Δ*tssL*(pTssL-His) grown in AB-MES (pH 5.5) for 6 h at 25°C were diluted (1× served as 0.5 µg/µl) and treated with (+) or without (−) calf intestinal alkaline phosphatase (CIAP). Western blot analysis of protein samples separated by 7% Phos-tag SDS-PAGE and examined with specific antibody against 6×His. Total protein isolated from Δ*tssL* mutant was a negative control. Phos-tag SDS-PAGE revealed the upper band indicating the phosphorylated TssL-His (*p*-TssL-His) and lower band indicating unphosphorylated TssL-His. (**C**) Phos-tag SDS-PAGE analysis for Fha. Western blot analysis of total protein isolated from wild-type C58 grown in AB-MES (pH 5.5) for 6 h at 25°C that was diluted (1× served as 0.5 µg/µl), treated with (+) or without (−) CIAP and separated by 7% Phos-tag SDS-PAGE and examined with specific antibodies against 6×His and Fha. TssL-His was pulled down by Ni-NTA binding beads and treated with (+) or without (−) CIAP as a positive control. Total protein isolated from Δ*t6* was a negative control. No phosphorylated Fha protein could be detected. The upper band detected in both C58 and Δ*t6* mutant was a non-specific cross-reacted protein as indicated.
(TIF)

Figure S4 **Sequence coverage and phosphorylated Thr 14 of TssL-His by Mass spectrometry identification.** Sequence coverage of TssL-His by MS/MS analysis is marked in red and the phosphorylated Thr 14 is indicated by a blue box. Thr 14 is the only phosphorylation site of TssL-His detected by MS from samples prepared by trypsin only or trypsin/chymotrypsin double digestion. Similar results were obtained from 3 independent experiments.
(TIF)

Figure S5 **Amino acid sequence alignment of PpkA orthologs.** Partial amino acid sequences of PpkA orthologs from *A. tumefaciens* C58 (Atu4330, GI:159186119), *P. aeruginosa* PAO1 (PA0074, GI:15595272), *Mycobacterium tuberculosis* UT205 (PknB, GI:378543286), and *Yersinia enterocolitica* (YpkA, GI: 1401295). Identical amino acid residues are highlighted in black. Amino acid residues used for mutagenesis are indicated with red asterisks. Sequences were aligned and highlighted by use of ClustalW2 (http://www.ebi.ac.uk/Tools/msa/clustalw2/).
(TIF)

Figure S6 **SDS-PAGE analysis of purified Fha proteins and isothermal titration calorimetry (ITC) analysis.** (**A**) Truncated Fha proteins tagged with 6×His were purified with Ni-NTA resins followed by HiLoad 16/600 Superdex75 pg column. Five µg of purified Fha proteins (Fha7-267^WT^, Fha7-267^R30AS46A^, Fha7-309^WT^) was analyzed by 12% SDS-PAGE and stained by Coomassie blue to ensure protein purity. Molecular markers are indicated on the left (kDa). (**B**) ITC of specific interaction and binding kinetics between the Fha7-309^WT^ protein and phosphorylated TssL peptide (DLPpTVVEI, *p*-TssL^8 mer^). The equilibrium dissociation constant (*K_d_*) between Fha7-309^WT^ protein and DLPpTVVEI (*p*-TssL^8 mer^) is 2.15±0.01 mM. The binding affinity of Fha7-309^WT^ protein and DLPTVVEI unphosphopeptide (TssL^8 mer^) is undetectable. Top panel shows the raw calorimetric data for the interaction and bottom panel the integrated heat changes, corrected for heat of dilution, and fitted to a single-site binding model.
(TIF)

Figure S7 **N-terminal amino acid sequence alignment of** ***A. tumefaciens*** **TssL with TssL orthologs encoded by closely related bacterial species.** Alignment of first 50 amino acid sequences of TssL orthologs identified from various *Agrobacterium* and *Rhizobia* strains were performed. The identical amino acid residues are highlighted in black and the conserved T14 residue is indicated with a red arrowhead. The analyzed proteins are TssL/Atu4333/OmpA-like porin [*Agrobacterium tumefaciens* C58]/(GI:159186121), OmpA-like porin [*A. tumefaciens* 5A]/(GI:418409003), OmpA-like porin [*A. tumefaciens* CCNWGS0286]/(GI:418297583), OmpA-like porin [*Agrobacterium* sp. ATCC 31749]/(GI:335037634), OmpA-like porin [*Agrobacterium* sp. H13-3]/(GI:332715427), OmpA-type porin [*Agrobacterium vitis* S4]/(GI:222106979), Type VI secretion system OmpA/MotB family protein [*Rhizobium leguminosarum bv. trifolii* WSM597]/(GI:424918217), Type VI secretion system OmpA/MotB family protein [*R. leguminosarum bv. trifolii* WSM2297]/(GI:424892095), Nitrogen fixation outer membrane porin [*R. leguminosarum bv. viciae* 3841]/(GI:116249130). Sequences were aligned and highlighted by use of ClustalW2 (http://www.ebi.ac.uk/Tools/msa/clustalw2/).
(TIF)

Figure S8 **Amino acid sequence alignment of** ***A. tumefaciens*** **TssL with TssL orthologs encoded by distantly related bacterial species.** Amino acid sequence of TssL orthologs from *A. tumefaciens* (Atu4333, GI:159186121), *Pseudomonas aeruginosa* PAO1 (PA0078, GI:15595276), *P. tolaasii* (TssL, GI: 515540981), *Pseudomonas fluorescens* (TssL, GI: 515541888), *Pseudomonas mandelii* (TssL, GI: 518410213), *Vibrio cholerae* O1 biovar EI Tor str. N16961 (VCA0115, GI:15600886), *Edwardsiella tarda* (EvpN, GI: 158512121), *Burkholderia mallei* (TssL, GI: 148750982), *Salmonella typhimurium* (SciP, GI: 15130931), *Burkholderia* sp. WSM4176 (TssL, GI: 517234167), *Acidovorax avenae* subsp. *avenae* ATCC 19860 (TssL, GI: 326316328), *Thiorhodococcus drewsii* (TssL, GI: 494100187), *Shewanella baltica* BA175 (TssL, GI: 386324502), enteroaggregative *Escherichia coli* Sci-1 T6SS (TssL, GI: 284924248), and *Francisella novicida* U112 (TssL/Ftn_1316, GI: 118497896). Part of the aligned result is shown here. Identical amino acid residues are highlighted in black. The T14 residue of TssL of *A. tumefaciens* is indicated with a red arrowhead. Sequences were aligned and highlighted by use of ClustalW2 (http://www.ebi.ac.uk/Tools/msa/clustalw2/).
(TIF)

Figure S9 **Secretion and western blot analyses assay on agar plate.** Western blot analysis of total and secreted (Sup) proteins isolated from various *A. tumefaciens* strains grown on agar plates were resolved by 10% or 12% Glycine-SDS-PAGE and examined with specific antibodies. ActC and RpoA were used as internal controls. The proteins analyzed are indicated on the left, and the molecular weight standards are indicated on the right and with arrows when necessary.
(TIF)

Figure S10 **Overexpression of TssL with T14A substitution in Δ** ***tssL*** **is unable to complement type VI secretion.** The Δ*tssL* mutant harboring the vector pRL662 (V) or complemented plasmid pTssL or pTssL^T14A^ was analyzed for Hcp and Atu4347 secretion. Western blot analysis of total (T) and secreted (S) proteins isolated from various strains grown in AB-MES (pH 5.5) for 6 h at 25°C and separated by 12% Glycine-SDS-PAGE and examined with specific antibodies. The non-secreted protein ActC and RpoA were internal controls for secretion and protein accumulation analyses, respectively. The proteins analyzed and the molecular weight standards are indicated on the left and right, respectively, and with arrows when necessary. The TssL with different migrations are marked with black asterisks.
(TIF)

Figure S11 **TssM is not required for TssL phosphorylation.** (**A**) Western blot analysis with regular SDS-PAGE of total proteins isolated from various *A. tumefaciens* strains examined with specific antibodies. RNA polymerase α subunit RpoA was an internal control. The TssL protein band with faster migration in Δ*ppkA* mutant is marked with an asterisk. (**B**) Phos-tag SDS-PAGE analysis. Western blot analysis of TssL-His proteins purified by Ni-NTA resins were separated by 7% Phos-tag SDS-PAGE and examined by specific antibody against 6×His. Total proteins from Δ*tssL* mutant were a negative control. Phos-tag SDS-PAGE revealed the upper band indicating phosphorylated TssL-His (*p*-TssL-His) and lower band indicating unphosphorylated TssL-His. The proteins analyzed and the molecular weight standards are indicated on the left and right, respectively.
(TIF)

Table S1 **Bacterial strains and plasmids.**
(PDF)

Table S2 **Primer information.**
(PDF)

Information S1 **Supporting Information.**
(PDF)
