# Supplementary figures and images for: Fha Interaction with Phosphothreonine of TssL Activates Type VI Secretion in Agrobacterium tumefaciens
Source: PLoS Pathog. 2014 Mar 13;10(3):e1003991. doi: 10.1371/journal.ppat.1003991 (PMC3953482; doi:10.1371/journal.ppat.1003991)

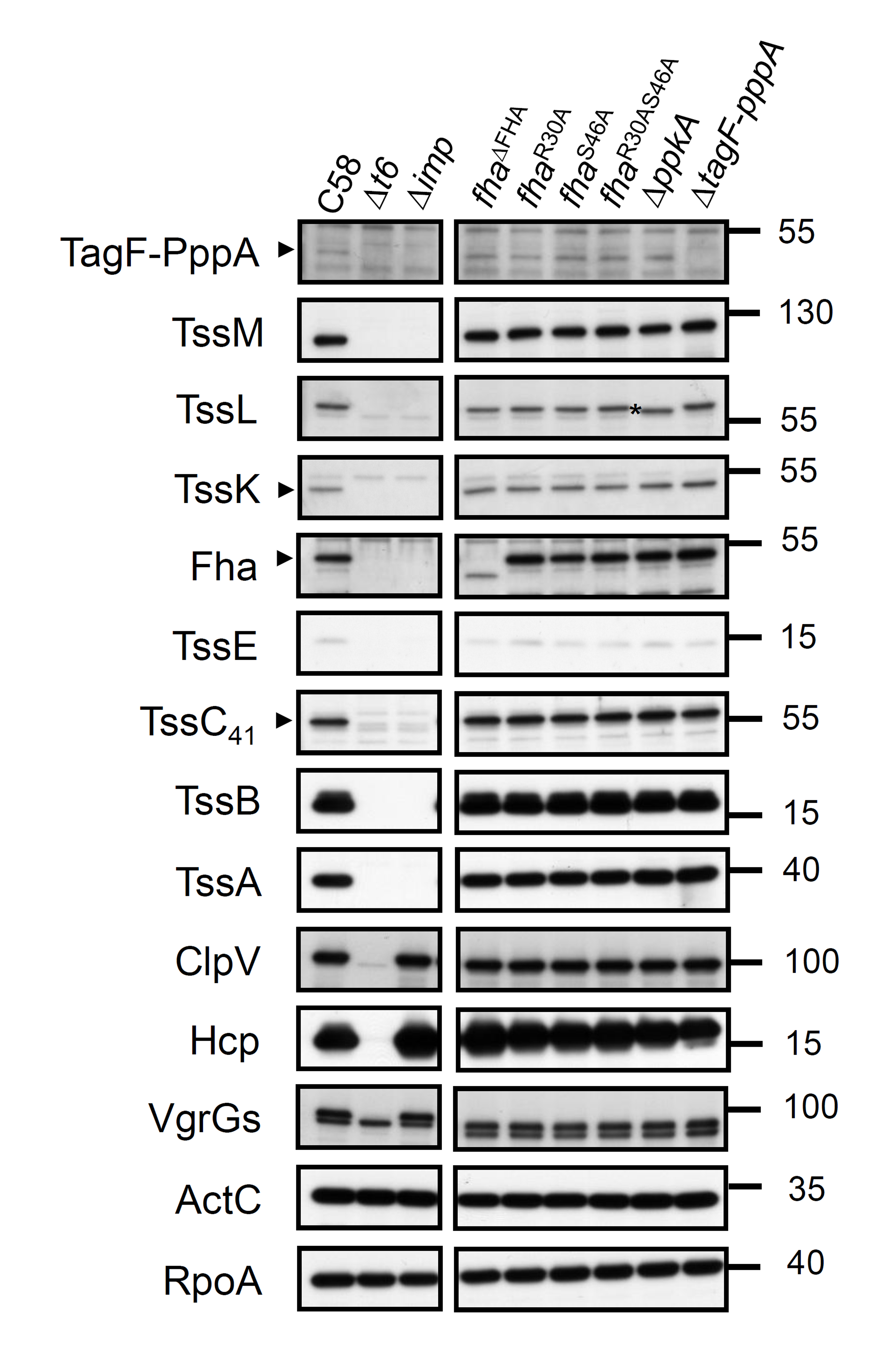

Supplement: Figure S1 — Western blot analysis of T6SS components in various strains. Western blot analysis of total proteins from various Agrobacterium tumefaciens strains resolved by 10% or 12% Glycine-SDS-PAGE and examined with specific antibodies. The soluble protein ActC and RNA polymerase α subunit (RpoA) were internal controls. The proteins analyzed are indicated on the left, the molecular weight standards are on the right, and with arrows when necessary. The TssL protein band with faster migration in ΔppkA mutant is marked with an asterisk. (TIF) [file ppat.1003991.s001.tif]

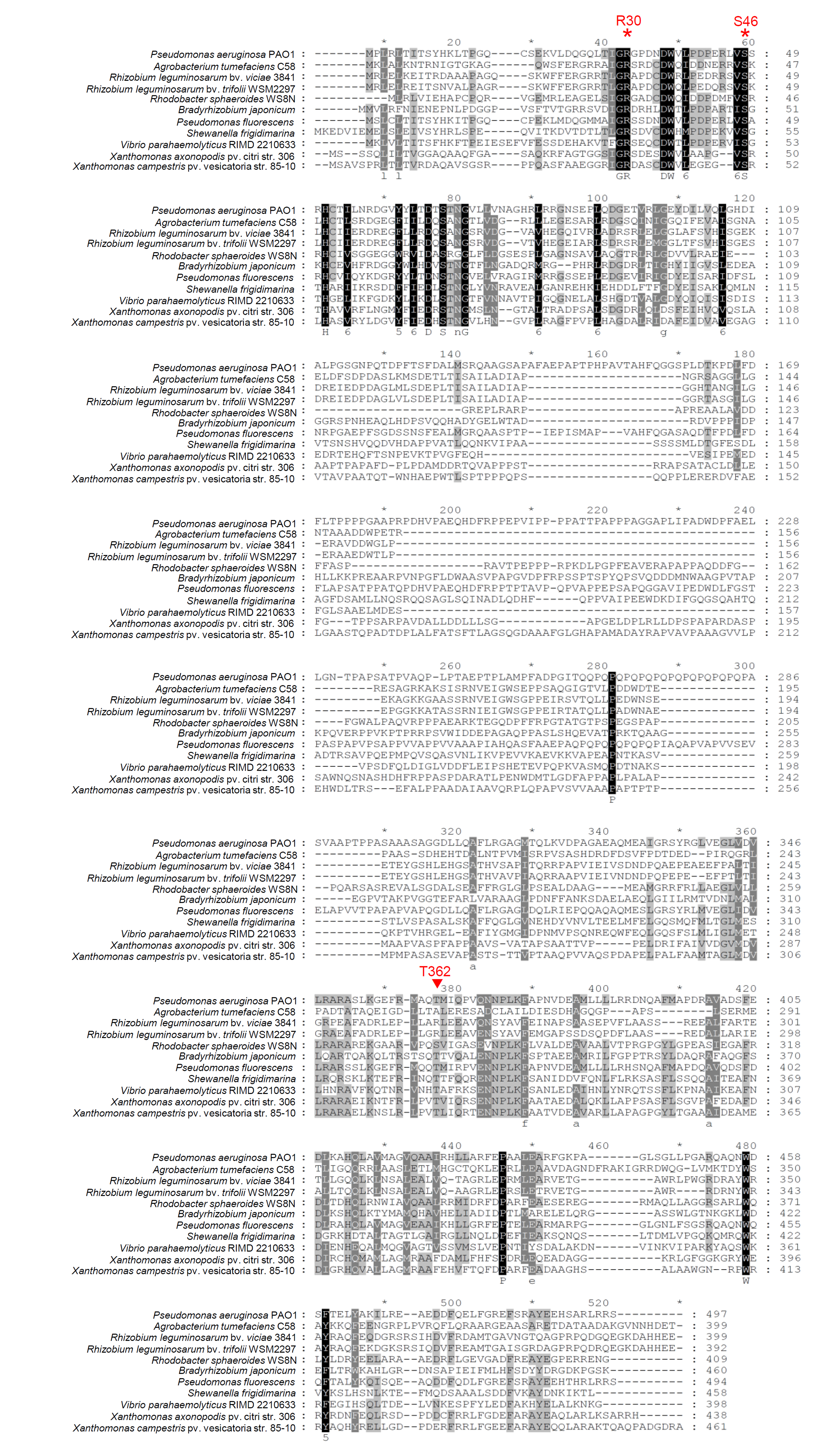

Supplement: Figure S2 — Alignment of the amino acid sequences of Fha orthologs. Amino acid sequences of Fha-family proteins from Pseudomonas aeruginosa (Fha1/PA0081, GI: 15595279), A. tumefaciens (Fha/Atu4335, GI: 15890648), Rhizobium leguminosarum bv. viciae 3841 (FHA domain-containing protein, GI: 115253782), R. leguminosarum bv. trifolii WSM2297 (FHA domain-containing protein, GI: 393183378), Rhodobacter sphaeroides WS8N (FHA domain-containing protein, GI: 332561141), Bradyrhizobium japonicum (Blr3598, GI: 27351858), Pseudomonas fluorescens (Fha, GI: 68347667), Shewanella frigidimarina (FHA domain-containing protein, GI: 122299446), Vibrio parahaemolyticus (Hypothetical protein, GI: 28809344), Xanthomonas axonopodis pv. citri (Conserved hypothetical protein, GI: 21110543), and Xanthomonas campestris pv. vesicatoria (Conserved hypothetical protein, GI: 78036121). Identical amino acid residues are highlighted in black. Conserved amino acid residues in FHA domain used for mutagenesis are indicated with red asterisks, and Thr 362 phosphorylation site of Fha1 of P. aeruginosa is indicated with an arrowhead. Sequences were aligned and highlighted by use of ClustalW2 (http://www.ebi.ac.uk/Tools/msa/clustalw2/). (TIF) [file ppat.1003991.s002.tif]

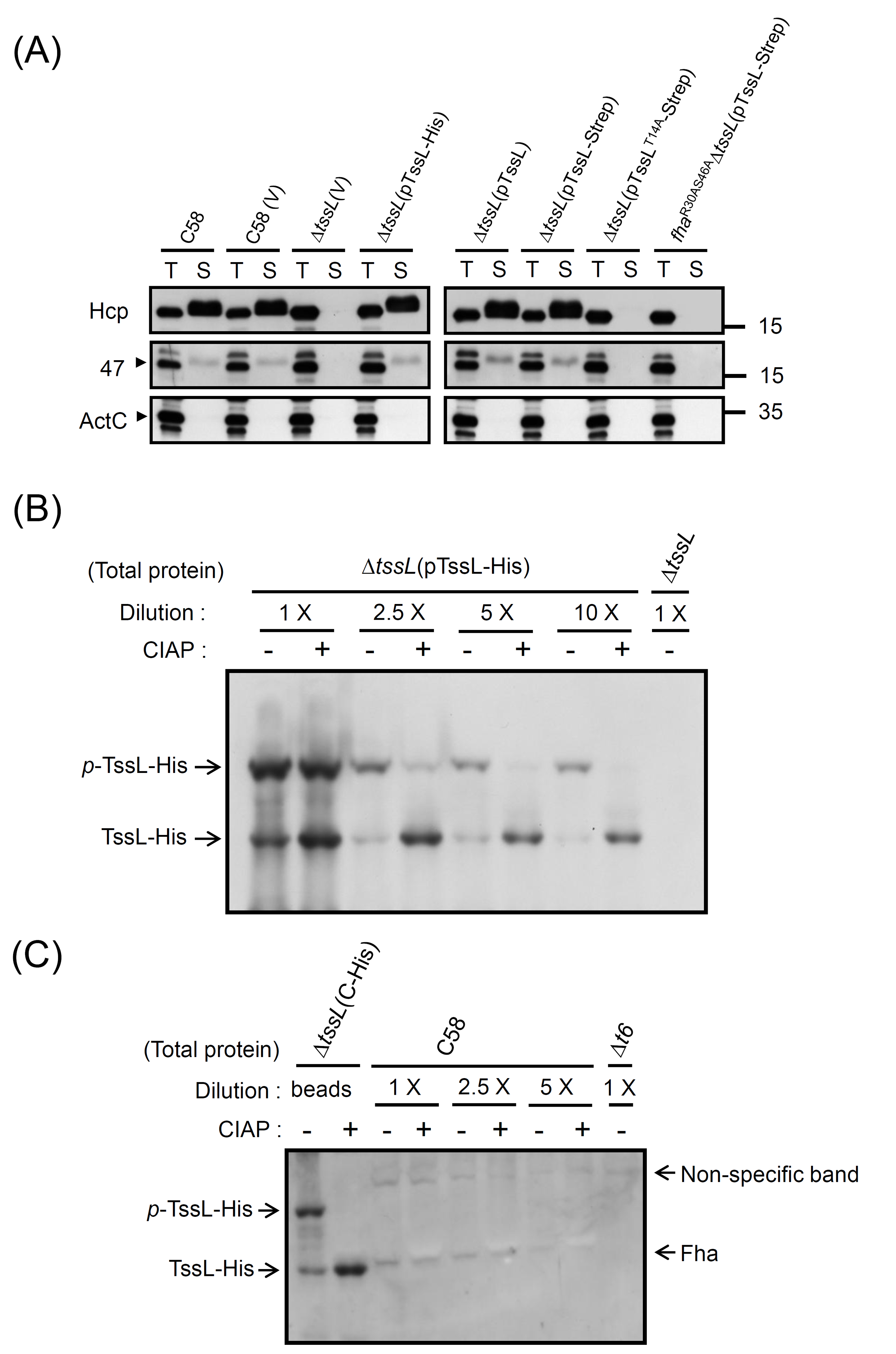

Supplement: Figure S3 — Protein secretion assay and Phos-tag SDS-PAGE analysis. (A) His-tagged or Strep-tagged TssL has full function in mediating Hcp and Atu4347 secretion. Western blot analysis of total (T) and secreted (S) protein isolated from various A. tumefaciens strains grown in AB-MES (pH 5.5) for 6 h at 25°C and separated by 12% Glycine-SDS-PAGE. The secreted proteins were collected from 1 ml of culture medium after removal of bacterial cells by centrifugation and were concentrated by TCA precipitation [41]. The non-secreted soluble protein ActC was an internal control. The proteins analyzed and molecular weight standards are on the left and right, respectively, and with arrows when necessary. (B) Phos-tag SDS-PAGE analysis of TssL-His. Total proteins isolated from ΔtssL(pTssL-His) grown in AB-MES (pH 5.5) for 6 h at 25°C were diluted (1× served as 0.5 µg/µl) and treated with (+) or without (−) calf intestinal alkaline phosphatase (CIAP). Western blot analysis of protein samples separated by 7% Phos-tag SDS-PAGE and examined with specific antibody against 6×His. Total protein isolated from ΔtssL mutant was a negative control. Phos-tag SDS-PAGE revealed the upper band indicating the phosphorylated TssL-His (p-TssL-His) and lower band indicating unphosphorylated TssL-His. (C) Phos-tag SDS-PAGE analysis for Fha. Western blot analysis of total protein isolated from wild-type C58 grown in AB-MES (pH 5.5) for 6 h at 25°C that was diluted (1× served as 0.5 µg/µl), treated with (+) or without (−) CIAP and separated by 7% Phos-tag SDS-PAGE and examined with specific antibodies against 6×His and Fha. TssL-His was pulled down by Ni-NTA binding beads and treated with (+) or without (−) CIAP as a positive control. Total protein isolated from Δt6 was a negative control. No phosphorylated Fha protein could be detected. The upper band detected in both C58 and Δt6 mutant was a non-specific cross-reacted protein as indicated. (TIF) [file ppat.1003991.s003.tif]

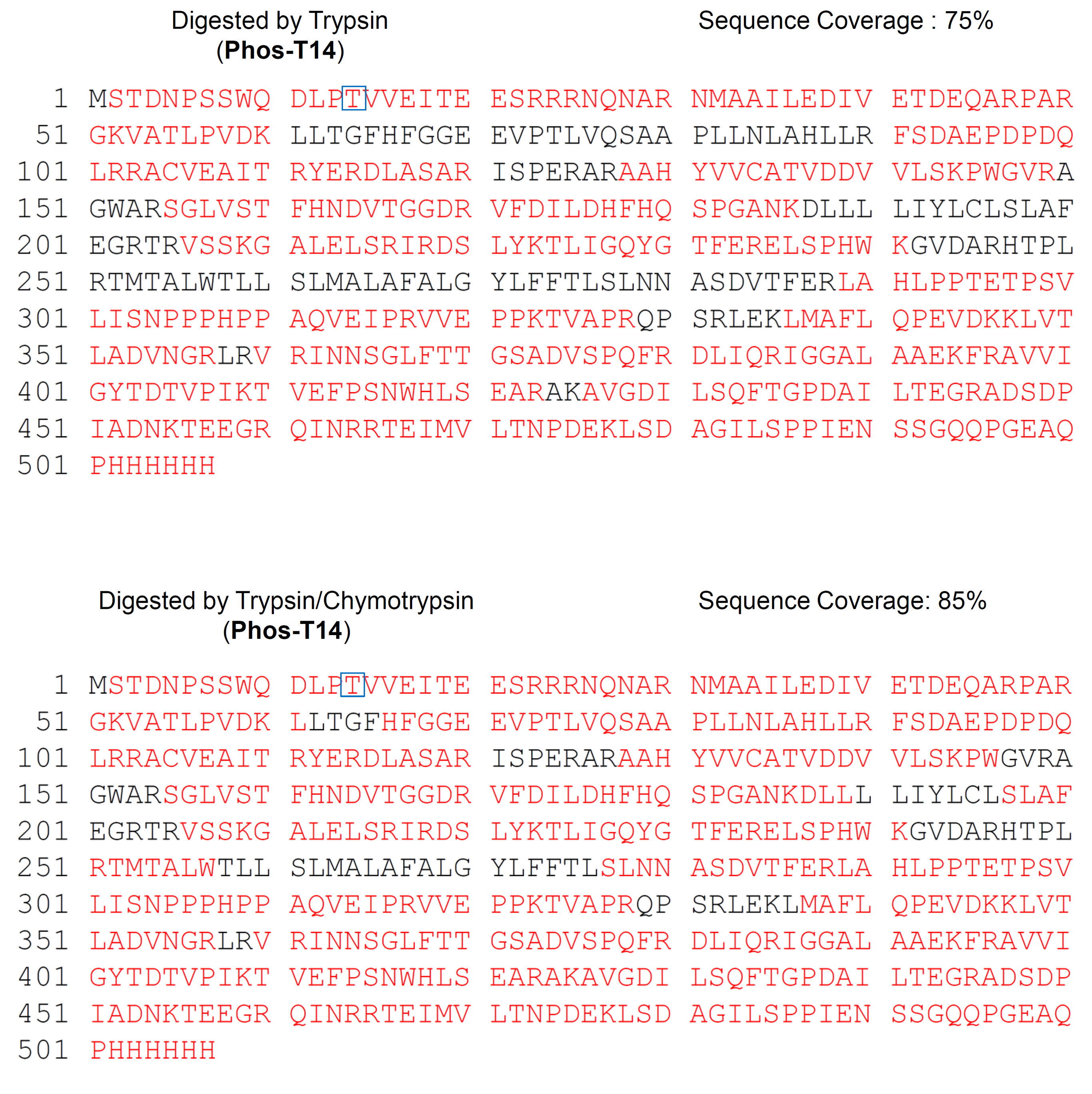

Supplement: Figure S4 — Sequence coverage and phosphorylated Thr 14 of TssL-His by Mass spectrometry identification. Sequence coverage of TssL-His by MS/MS analysis is marked in red and the phosphorylated Thr 14 is indicated by a blue box. Thr 14 is the only phosphorylation site of TssL-His detected by MS from samples prepared by trypsin only or trypsin/chymotrypsin double digestion. Similar results were obtained from 3 independent experiments. (TIF) [file ppat.1003991.s004.tif]

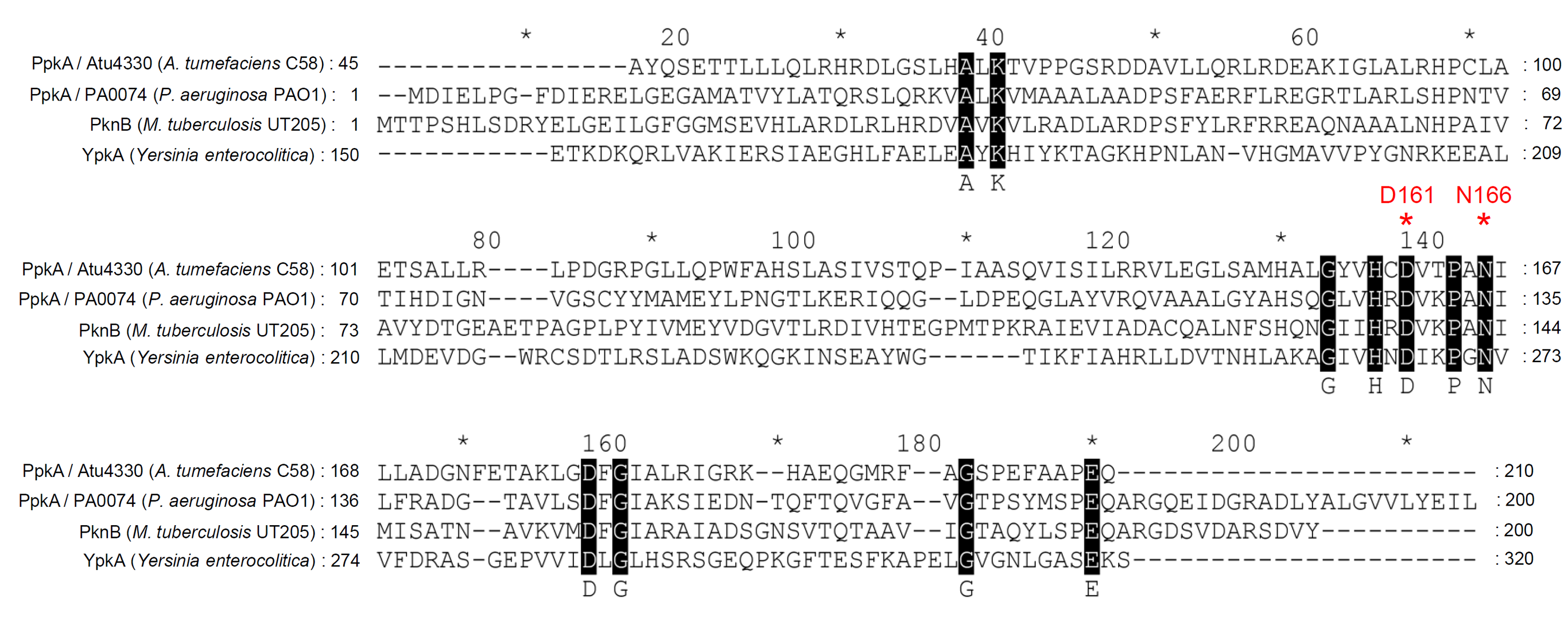

Supplement: Figure S5 — Amino acid sequence alignment of PpkA orthologs. Partial amino acid sequences of PpkA orthologs from A. tumefaciens C58 (Atu4330, GI:159186119), P. aeruginosa PAO1 (PA0074, GI:15595272), Mycobacterium tuberculosis UT205 (PknB, GI:378543286), and Yersinia enterocolitica (YpkA, GI: 1401295). Identical amino acid residues are highlighted in black. Amino acid residues used for mutagenesis are indicated with red asterisks. Sequences were aligned and highlighted by use of ClustalW2 (http://www.ebi.ac.uk/Tools/msa/clustalw2/). (TIF) [file ppat.1003991.s005.tif]

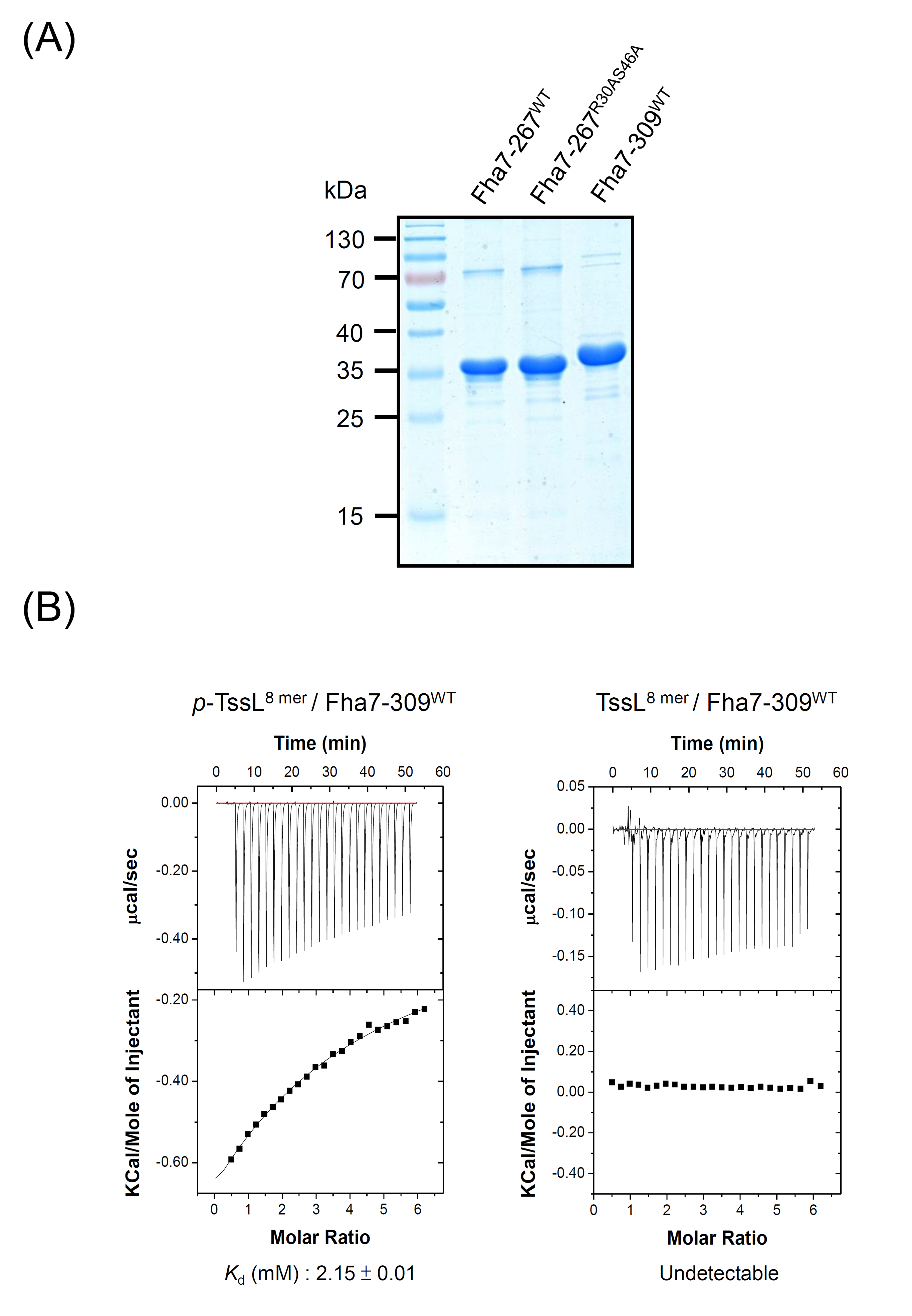

Supplement: Figure S6 — SDS-PAGE analysis of purified Fha proteins and isothermal titration calorimetry (ITC) analysis. (A) Truncated Fha proteins tagged with 6×His were purified with Ni-NTA resins followed by HiLoad 16/600 Superdex75 pg column. Five µg of purified Fha proteins (Fha7-267WT, Fha7-267R30AS46A, Fha7-309WT) was analyzed by 12% SDS-PAGE and stained by Coomassie blue to ensure protein purity. Molecular markers are indicated on the left (kDa). (B) ITC of specific interaction and binding kinetics between the Fha7-309WT protein and phosphorylated TssL peptide (DLPpTVVEI, p-TssL8 mer). The equilibrium dissociation constant (Kd) between Fha7-309WT protein and DLPpTVVEI (p-TssL8 mer) is 2.15±0.01 mM. The binding affinity of Fha7-309WT protein and DLPTVVEI unphosphopeptide (TssL8 mer) is undetectable. Top panel shows the raw calorimetric data for the interaction and bottom panel the integrated heat changes, corrected for heat of dilution, and fitted to a single-site binding model. (TIF) [file ppat.1003991.s006.tif]

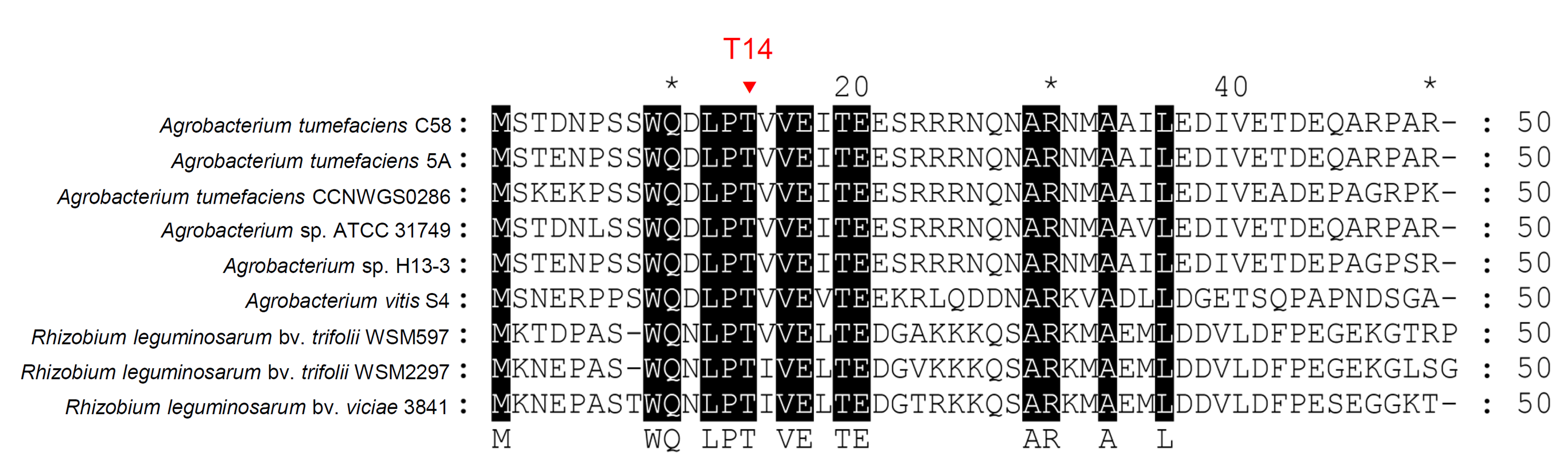

Supplement: Figure S7 — N-terminal amino acid sequence alignment of A. tumefaciens TssL with TssL orthologs encoded by closely related bacterial species. Alignment of first 50 amino acid sequences of TssL orthologs identified from various Agrobacterium and Rhizobia strains were performed. The identical amino acid residues are highlighted in black and the conserved T14 residue is indicated with a red arrowhead. The analyzed proteins are TssL/Atu4333/OmpA-like porin [Agrobacterium tumefaciens C58]/(GI:159186121), OmpA-like porin [A. tumefaciens 5A]/(GI:418409003), OmpA-like porin [A. tumefaciens CCNWGS0286]/(GI:418297583), OmpA-like porin [Agrobacterium sp. ATCC 31749]/(GI:335037634), OmpA-like porin [Agrobacterium sp. H13-3]/(GI:332715427), OmpA-type porin [Agrobacterium vitis S4]/(GI:222106979), Type VI secretion system OmpA/MotB family protein [Rhizobium leguminosarum bv. trifolii WSM597]/(GI:424918217), Type VI secretion system OmpA/MotB family protein [R. leguminosarum bv. trifolii WSM2297]/(GI:424892095), Nitrogen fixation outer membrane porin [R. leguminosarum bv. viciae 3841]/(GI:116249130). Sequences were aligned and highlighted by use of ClustalW2 (http://www.ebi.ac.uk/Tools/msa/clustalw2/). (TIF) [file ppat.1003991.s007.tif]

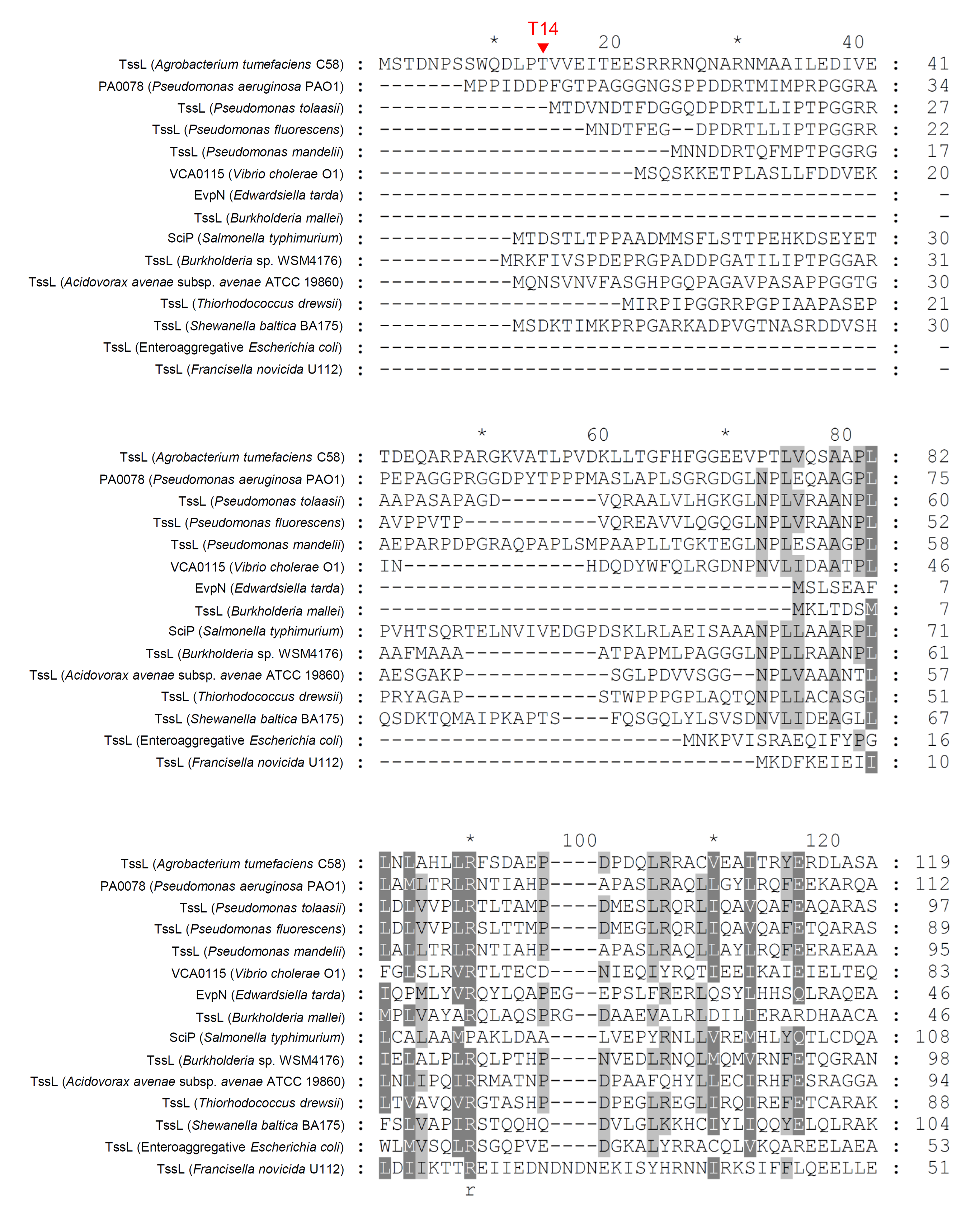

Supplement: Figure S8 — Amino acid sequence alignment of A. tumefaciens TssL with TssL orthologs encoded by distantly related bacterial species. Amino acid sequence of TssL orthologs from A. tumefaciens (Atu4333, GI:159186121), Pseudomonas aeruginosa PAO1 (PA0078, GI:15595276), P. tolaasii (TssL, GI: 515540981), Pseudomonas fluorescens (TssL, GI: 515541888), Pseudomonas mandelii (TssL, GI: 518410213), Vibrio cholerae O1 biovar EI Tor str. N16961 (VCA0115, GI:15600886), Edwardsiella tarda (EvpN, GI: 158512121), Burkholderia mallei (TssL, GI: 148750982), Salmonella typhimurium (SciP, GI: 15130931), Burkholderia sp. WSM4176 (TssL, GI: 517234167), Acidovorax avenae subsp. avenae ATCC 19860 (TssL, GI: 326316328), Thiorhodococcus drewsii (TssL, GI: 494100187), Shewanella baltica BA175 (TssL, GI: 386324502), enteroaggregative Escherichia coli Sci-1 T6SS (TssL, GI: 284924248), and Francisella novicida U112 (TssL/Ftn_1316, GI: 118497896). Part of the aligned result is shown here. Identical amino acid residues are highlighted in black. The T14 residue of TssL of A. tumefaciens is indicated with a red arrowhead. Sequences were aligned and highlighted by use of ClustalW2 (http://www.ebi.ac.uk/Tools/msa/clustalw2/). (TIF) [file ppat.1003991.s008.tif]

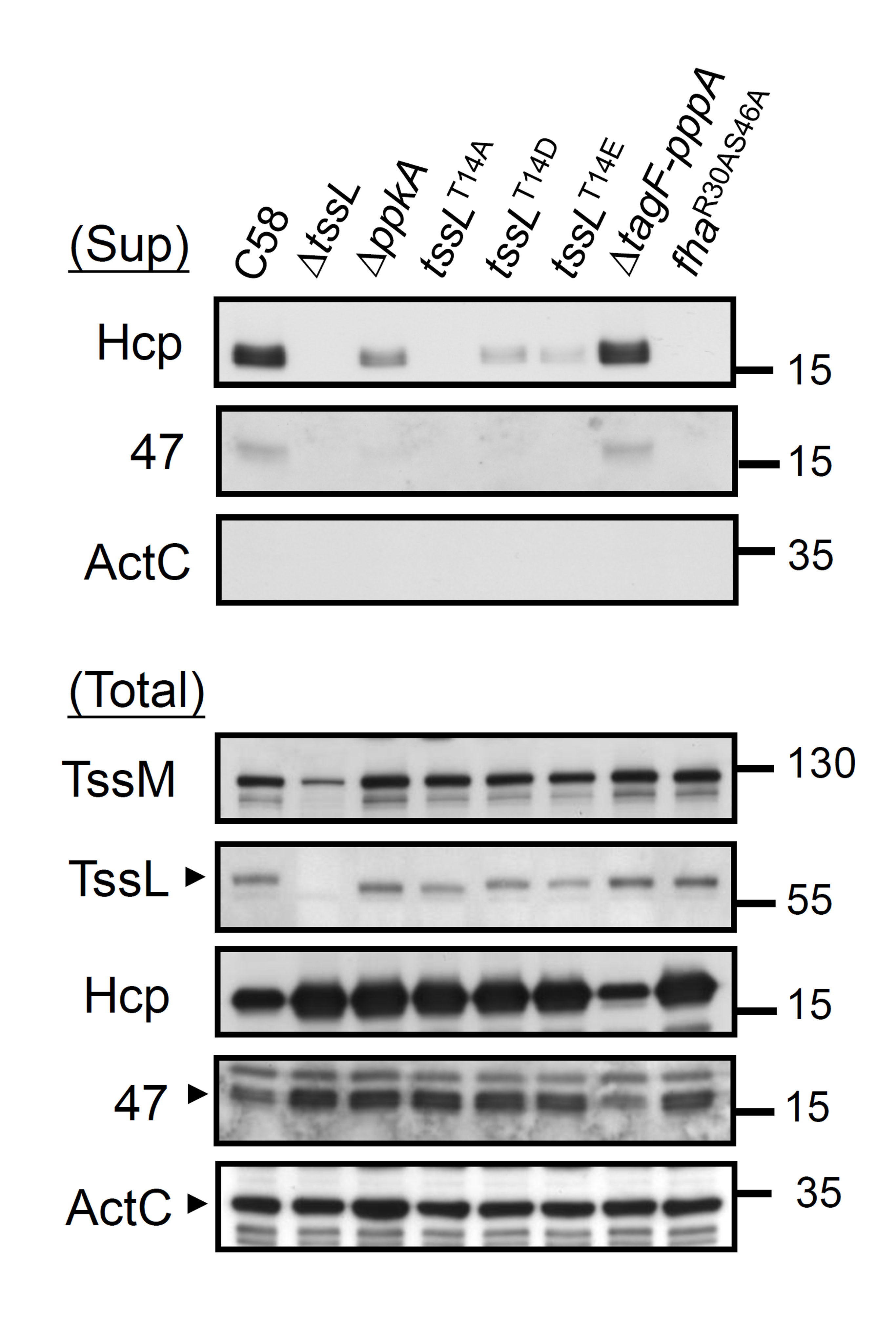

Supplement: Figure S9 — Secretion and western blot analyses assay on agar plate. Western blot analysis of total and secreted (Sup) proteins isolated from various A. tumefaciens strains grown on agar plates were resolved by 10% or 12% Glycine-SDS-PAGE and examined with specific antibodies. ActC and RpoA were used as internal controls. The proteins analyzed are indicated on the left, and the molecular weight standards are indicated on the right and with arrows when necessary. (TIF) [file ppat.1003991.s009.tif]

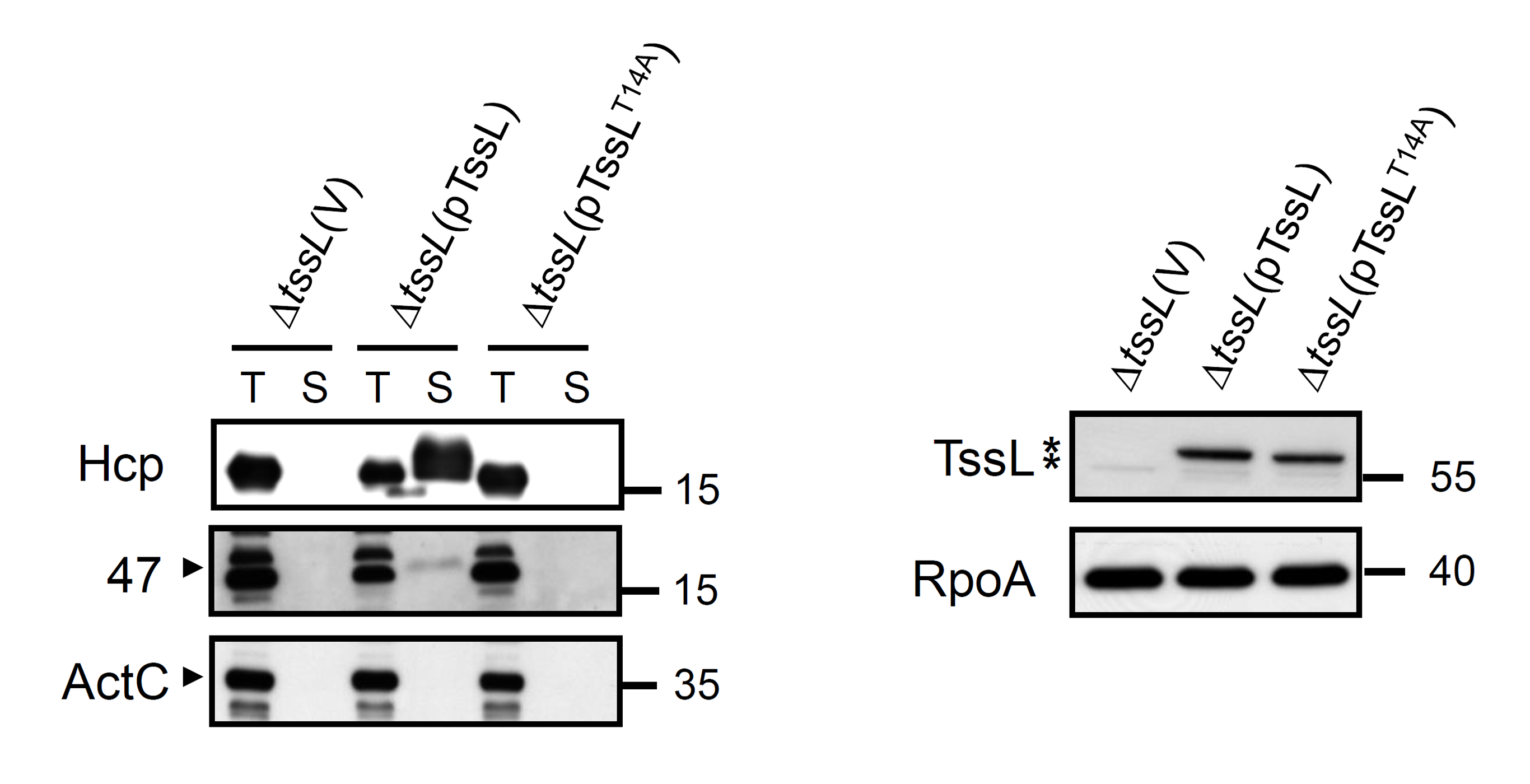

Supplement: Figure S10 — Overexpression of TssL with T14A substitution in Δ tssL is unable to complement type VI secretion. The ΔtssL mutant harboring the vector pRL662 (V) or complemented plasmid pTssL or pTssLT14A was analyzed for Hcp and Atu4347 secretion. Western blot analysis of total (T) and secreted (S) proteins isolated from various strains grown in AB-MES (pH 5.5) for 6 h at 25°C and separated by 12% Glycine-SDS-PAGE and examined with specific antibodies. The non-secreted protein ActC and RpoA were internal controls for secretion and protein accumulation analyses, respectively. The proteins analyzed and the molecular weight standards are indicated on the left and right, respectively, and with arrows when necessary. The TssL with different migrations are marked with black asterisks. (TIF) [file ppat.1003991.s010.tif]

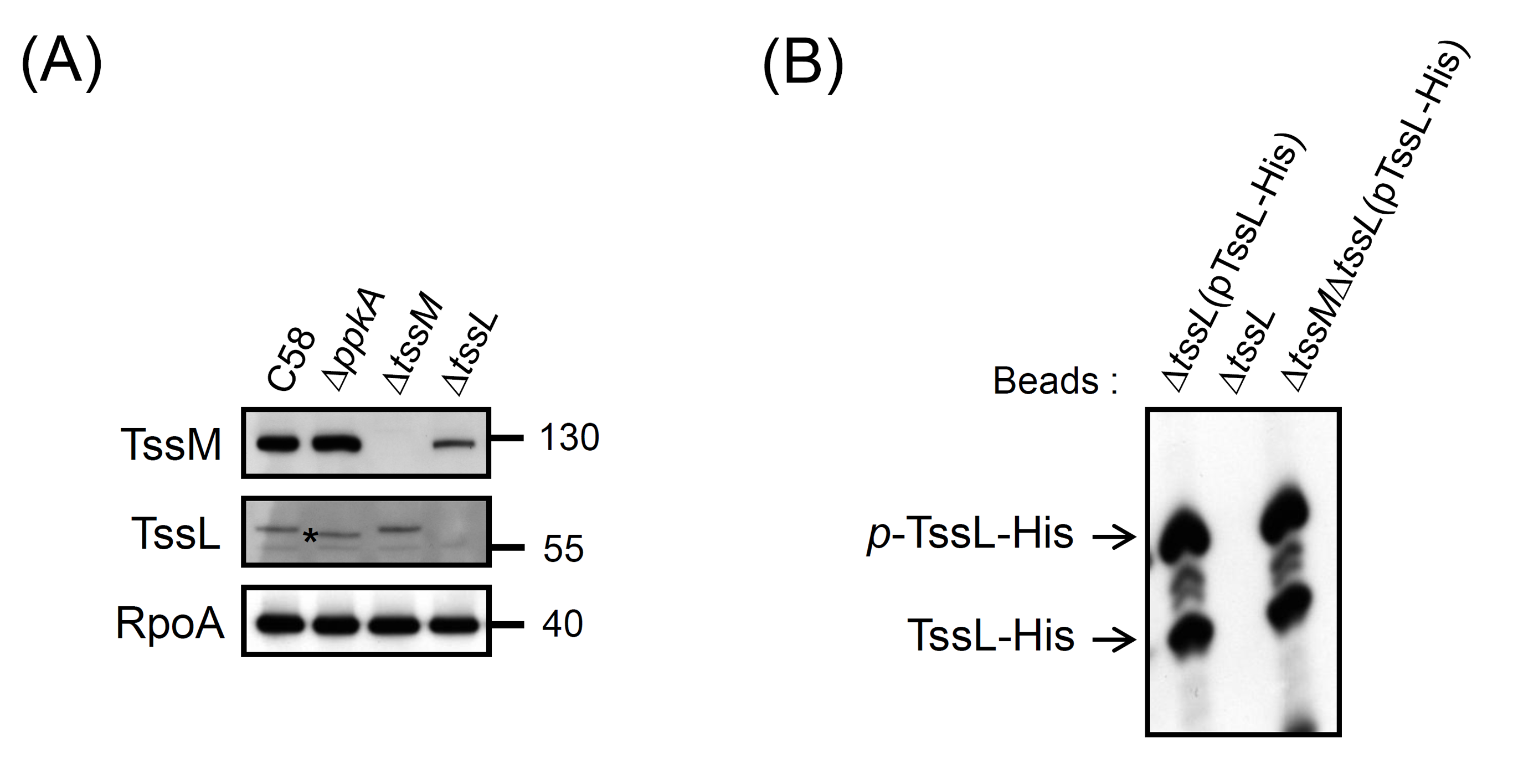

Supplement: Figure S11 — TssM is not required for TssL phosphorylation. (A) Western blot analysis with regular SDS-PAGE of total proteins isolated from various A. tumefaciens strains examined with specific antibodies. RNA polymerase α subunit RpoA was an internal control. The TssL protein band with faster migration in ΔppkA mutant is marked with an asterisk. (B) Phos-tag SDS-PAGE analysis. Western blot analysis of TssL-His proteins purified by Ni-NTA resins were separated by 7% Phos-tag SDS-PAGE and examined by specific antibody against 6×His. Total proteins from ΔtssL mutant were a negative control. Phos-tag SDS-PAGE revealed the upper band indicating phosphorylated TssL-His (p-TssL-His) and lower band indicating unphosphorylated TssL-His. The proteins analyzed and the molecular weight standards are indicated on the left and right, respectively. (TIF) [file ppat.1003991.s011.tif]
