# Supplementary material for: Fha Interaction with Phosphothreonine of TssL Activates Type VI Secretion in Agrobacterium tumefaciens
Source: PLoS Pathog. 2014 Mar 13;10(3):e1003991. doi: 10.1371/journal.ppat.1003991 (PMC3953482; doi:10.1371/journal.ppat.1003991)
Supplement: Table S1 — Bacterial strains and plasmids. (PDF) [file ppat.1003991.s012.pdf]

Table S1. Bacterial strains and plasmids

Strain /plasmid	Relevant characteristics	Source/ reference
A. tumefaciens		
C58	Wild type virulent strain containing nopaline-type Ti plasmid pTiC58	Eugene Nester
EML3685	Entire *t6ss* gene cluster deletion mutant, C58Δ*t6*	This study
EML1218	Entire *imp* operon deletion mutant, C58Δ*imp*	[4]
EML1060	*ppkA*(*atu4330*) in frame deletion mutant, C58Δ*ppkA*	[4]
EML1063	*tagF-pppA* (*atu4331*) in frame deletion mutant, C58Δ*tagF-pppA*	[4]
EML1068	*tssM* (*atu4332*) in-frame deletion mutant, C58Δ*tssM*	[3]
EML1073	*tssL* (*atu4333*) in-frame deletion mutant, C58Δ*tssL*	[3]
EML1761	*tssM* and *tssL* in frame deletion mutant, C58Δ*tssM*Δ*tssL*	[5]
EML1521	*fha* (*atu4335*) in frame deletion mutant, C58Δ*fha*	[4]
EML3679	*fha* with deletion of entire FHA domain (25-76 a. a.), C58*fha*$^{\Delta FHA}$	This study
EML3694	*fha* with R30A substitution, C58*fha*^{R30A}	This study
EML3696	*fha* with S46A substitution, C58*fha*^{S46A}	This study
EML3698	*fha* with both R30A and S46A substitutions, C58*fha*^{R30AS46A}	This study
EML3706	*tssL* in-frame deletion mutant, and *fha* with both R30A and S46A substitutions, C58 *fha*^{R30AS46A}Δ*tssL*	This study
EML3703	*ppkA* and *tssL* in frame deletion mutant, C58Δ*ppkA*Δ*tssL*	This study
EML4163	*ppkA* with both D161A and N166A substitutions, C58*ppkA*^{D161AN166A}	This study
EML4166	*tssL* in-frame deletion mutant, and *ppkA* with both D161A and N166A substitutions, C58*ppkA*^{D161AN166A}Δ*tssL*	This study
EML3709	*tssL* with T14A substitution, C58*tssL*^{T14A}	This study
EML3855	*tssL* with T14D substitution, C58*tssL*^{T14D}	This study
EML3858	*tssL* with T14E substitution, C58*tssL*^{T14E}	This study
EML2137	Complementation of *fha* gene to linear chromosome of Δ*fha* strain, revertant strain of Δ*fha*	[4]
E. coli		
DH10B	Host for DNA cloning	Invitrogen
BL21(DE3)	Host for overexpressing proteins driven by T7 promoter	[6]
Plasmids		
pRL662	Gm^r, broad-host range vector derived from pBBR1MCS-2	[2]

pET22b(+)	Ap^r, *E. coli* overexpression vector to generate C-terminal His-tagged protein	Novagen
pJQ200KS	Gm^r, suicide plasmid containing Gm^r and *sacB* gene for selection of double crossover	[1]
pPpkA	Gm^r, pRL662 expressing PpkA driven by *lacZp*	This study
pTagF-PppA	Gm^r, pRL662 expressing TagF-PppA driven by *lacZp*	This study
pTssL	Gm^r, pRL662 expressing TssL driven by *lacZp*	[3]
pTssL-His	Gm^r, pRL662 expressing TssL-His fusion protein driven by *lacZp*	This study
pTssL-Strep	Gm^r, pRL662 expressing TssL-Strep fusion protein driven by *lacZp*	This study
pTssL^{T14A}	Gm^R, pRL662 expressing TssL with T14A substitution driven by *lacZp*	This study
pTssL^{T14A}-His	Gm^R, pRL662 expressing TssL-His fusion protein with T14A substitution driven by *lacZp*	This study
pTssL^{T14A}-Strep	Gm^R, pRL662 expressing TssL-Strep fusion protein with T14A substitution driven by *lacZp*	This study
pET-Fha7-267^{WT} -His	Ap^r, pET22b overexpressing His-tagged Fha (7-267 a.a.) in *E. coli*	This study
pET-Fha7-267^{R30AS46A}-His	Ap^r, pET22b overexpressing His-tagged Fha (7-267 a.a.) with both R30A and S46A substitutions in *E. coli*	This study
pET-Fha7-309^{WT} -His	Ap^r, pET22b overexpressing His-tagged Fha (7-309 a.a.) in *E. coli*	This study
pJQ200KS-Δ*t6*	Gm^r, used in generating entire *t6ss* gene cluster deletion mutant of *A. tumefaciens* C58	This study
pJQ200KS-*fha*^{ΔFHA}	Gm^r, used in generating *fha* with deletion of entire FHA domain (25-76 a. a.) of *A. tumefaciens* C58	This study
pJQ200KS- *fha*^{R30A}	Gm^r, used in generating *fha* with R30A substitution of *A. tumefaciens* C58	This study
pJQ200KS- *fha*^{S46A}	Gm^r, used in generating *fha* with S46A substitution of *A. tumefaciens* C58	This study
pJQ200KS- *fha*^{R30AS46A}	Gm^r, used in generating *fha* with both R30A and S46A substitutions of *A. tumefaciens* C58	This study

pJQ200KS- *tssL*^{T14A}	Gm^r, used in generating *tssL* with T14A substitution of *A. tumefaciens* C58	This study
pJQ200KS- *tssL*^{T14D}	Gm^r, used in generating *tssL* with T14D substitution of *A. tumefaciens* C58	This study
pJQ200KS- *tssL*^{T14E}	Gm^r, used in generating *tssL* with T14E substitution of *A. tumefaciens* C58	This study
pJQ200KS-*ppkA*^{D161AN166A}	Gm^r, used in generating *ppkA* with both D161A and N166A substitutions of *A. tumefaciens* C58	This study

References

1. Quandt J, Hynes MF (1993) Versatile suicide vectors which allow direct selection for gene replacement in gram-negative bacteria. Gene 127: 15-21.
2. Vergunst AC, Schrammeijer B, den Dulk-Ras A, de Vlaam CM, Regensburg-Tuink TJ, et al. (2000) VirB/D4-dependent protein translocation from *Agrobacterium* into plant cells. Science 290: 979-982.
3. Ma LS, Lin JS, Lai EM (2009) An IcmF family protein, $ImpL_M$, is an integral inner membrane protein interacting with $ImpK_L$, and its walker a motif is required for type VI secretion system-mediated Hcp secretion in *Agrobacterium tumefaciens*. J Bacteriol 191: 4316-4329.
4. Lin JS, Ma LS, Lai EM (2013) Systematic Dissection of the *Agrobacterium* Type VI Secretion System Reveals Machinery and Secreted Components for Subcomplex Formation. PLoS One 8: e67647.
5. Ma LS, Narberhaus F, Lai EM (2012) IcmF family protein TssM exhibits ATPase activity and energizes type VI secretion. J Biol Chem 287: 15610-15621.
6. Studier FW, Rosenberg AH, Dunn JJ, Dubendorff JW (1990) Use of T7 RNA polymerase to direct expression of cloned genes. Methods Enzymol 185: 60-89.
