# Supplementary material for: Fha Interaction with Phosphothreonine of TssL Activates Type VI Secretion in Agrobacterium tumefaciens
Source: PLoS Pathog. 2014 Mar 13;10(3):e1003991. doi: 10.1371/journal.ppat.1003991 (PMC3953482; doi:10.1371/journal.ppat.1003991)
Supplement: Table S2 — Primer information. (PDF) [file ppat.1003991.s013.pdf]

Table S2. Primer information

Primer	Plasmid	Sequence (5 '-3')[a]	Source / reference
PpkA F-XhoI	pPpkA	5'-CCGCTCGAGAGGTAGCCGGACGTTTTTCC-3'	This study
PpkA R-XbaI		5'-GCTCTAGACGTGTTCCAGAAGAGTGTGC-3'	This study
PppA F-BamHI	pPppA	5'-CGGGATCCTTATGATGAAGGCAAGCACG-3'	This study
PppA R-XbaI		5'-GCTCTAGAGGATAGGCAGGCTCATCAAG-3'	This study
TssL F-XhoI	1. pTssL-His (TssL F-XhoI/TssL-His R-XbaI)<br>2. pTssL-Strep (TssL F-XhoI/TssL-Strep R-XbaI)<br>3. pTssL^{T14A}-His (pJQ200KS- *tssL*^{T14A} as template) (TssL F-XhoI/TssL-His R-XbaI)<br>4. pTssL^{T14A}-Strep (pJQ200KS-*tssL*^{T14A} as template) (TssL F-XhoI/TssL-Strep R-XbaI)	5'-CCGCTCGAGTGCCATTGCCCTGCATCTGG-3'	[1]
TssL-His R-XbaI		5'-GCTCTAGATCAGTGGTGGTGGTGGTGGTGTGGCTGGGCCTCTCCCGGCT-3'	This study
TssL-Strep R-XbaI		5'-GCTCTAGATCACTTTTCGAACTGCGGGTGGCTCCATGGCTGGGCCTCTCCCGGCTG-3'	This study
TssL F-XhoI	pTssL^{T14A} (pJQ200KS-*tssL*^{T14A} as template)	5'-CCGCTCGAGTGCCATTGCCCTGCATCTGG-3'	[1]
TssL R-XbaI		5'-GCTCTAGATCTCGACATAGGAACGGATCG-3'	[1]
Fha F-NdeI	1. pET-Fha7-267^{WT}–His<br>2. pET-Fha7-267^{R30AS46A}-His (pJQ200KS-*fha*^{R30AS46A} as template)	5'-GAACATATGAACACACGGAACATCGGGAC-3'	This study
Fha^{267} R-XhoI		5'-CCGCTCGAGGCAATCGGCACTTTCCCTTTC-3'	This study
Fha F-NdeI	pET-Fha7-309^{WT}-His	5'-GAACATATGAACACACGGAACATCGGGAC-3'	This study
Fha^{309} R-XhoI		5'-CCGCTCGAGATGCATGAGGGTTTCGAGC-3'	This study
PpkA 2F-BamHI	pJQ200KS-Δ*t6*	5'-CGGGATCCCTGTAGCGCCGGCGTCAGTTG-3'	[2]
PpkA 2R-XmaI		5'-TCCCCCCGGGCCCGTCAGGAGCGTGTACTTG-3'	[2]
Atu4352 2F-BamHI		5'-CGGGATCCGAGTGACGACGATATCCAGC-3'	[2]
Atu4352 2R-XbaI		5'-GCTCTAGACTCGATCTTGAAATCACCGAG-3'	[2]
Fha 1F-XbaI	pJQ200KS-*fha*$^{\Delta FHA}$	5'-GCTCTAGATGCCGAAGACACACTTCTGC-3'	This study
Fha 2R-XmaI		5'-TCCCCCCGGGGGAGGAATGAAATCCGGATCG-3'	This study
Fha$^{\Delta FHA}$-1		5'-GACGCGCGCTTTCGCCCTCAAGGCCTCGCTCGAAGCTCCACTG-3'	This study
Fha$^{\Delta FHA}$-2		5'-CAGTGGAGCTTCGAGCGAGGCCTTGAGGGCGAAAGCGCGCGTC-3'	This study

Fha 1F-XbaI	pJQ200KS-*fha*^{R30A}	5'-GCTCTAGATGCCGAAGACACACTTCTGC-3'	This study
Fha 2R-XmaI		5'-TCCCCCCGGGGGAGGAATGAAATCCGGATCG-3'	This study
Fha^{R30A}-1		5'-CAGTCGCAATCACGAGA**CGC**GCCAATTGCGCGGCGGCCTC-3'	This study
Fha^{R30A}-2		5'-CCGCCGCGCAATTGGC**GCG**TCTCGTGATTGCGACTGGCAG-3'	This study
Fha 1F-XbaI	1. pJQ200KS-*fha*^{S46A} 2. pJQ200KS-*fha*^{R30AS46A} (pJQ200KS-*fha*^{R30A} as template)	5'-GCTCTAGATGCCGAAGACACACTTCTGC-3'	This study
Fha 2R-XmaI		5'-TCCCCCCGGGGGAGGAATGAAATCCGGATCG-3'	This study
Fha^{S46A}-1		5'-GCGTGCAATGCAGCTT**GGC**AACGCGCCGCTCATTGTCATC-3'	This study
Fha^{S46A}-2		5'-ACAATGAGCGGCGCGTT**GCC**AAGCTGCATTGCACGCTGAG-3'	This study
TssL 1F-XbaI	pJQ200KS- *tssL*^{T14A}	5'-GCTCTAGACGAAGACTGCATCCAGCTTGC-3'	[1]
TssL 2R-XmaI		5'-TCCCCCCGGGAGTGTCGATAAGGATCGCCTC-3'	[1]
TssL^{T14A}-1		5'-CCTCGGTGATCTCGACCAC**CGC**CGGCAAATCCTGCCAGG-3'	This study
TssL^{T14A}-2		5'-CCTGGCAGGATTTGCCG**GCG**GTGGTCGAGATCACCGAGG-3'	This study
TssL 1F-XbaI	pJQ200KS- *tssL*^{T14D}	5'-GCTCTAGACGAAGACTGCATCCAGCTTGC-3'	[1]
TssL 2R-XmaI		5'-TCCCCCCGGGAGTGTCGATAAGGATCGCCTC-3'	[1]
TssL^{T14D}-1		5'-CCTCGGTGATCTCGACCAC**ATC**CGGCAAATCCTGCCAGG-3'	This study
TssL^{T14D}-2		5'-CCTGGCAGGATTTGCCG**GAT**GTGGTCGAGATCACCGAGG-3'	This study
TssL 1F-XbaI	pJQ200KS- *tssL*^{T14E}	5'-GCTCTAGACGAAGACTGCATCCAGCTTGC-3'	[1]
TssL 2R-XmaI		5'-TCCCCCCGGGAGTGTCGATAAGGATCGCCTC-3'	[1]
TssL^{T14E}-1		5'-CCTCGGTGATCTCGACCAC**CTC**CGGCAAATCCTGCCAGG-3'	This study
TssL^{T14E}-2		5'-CCTGGCAGGATTTGCCG**GAG**GTGGTCGAGATCACCGAGG-3'	This study
PpkA 1F-XbaI	pJQ200KS-*ppkA*^{D161AN166A}	5'-GCTCTAGAGGAGATGATGGCACAGCAGATC-3'	This study
PpkA 2R-XmaI		5'-TCCCCCCGGGCCCGTCAGGAGCGTGTACTTG-3'	This study
PpkA^{D161AN166A}-1		5'-CATCCGCCAGCAGAAT**AGC**GGCTGGCGTGAC**GGC**GCAATGCACATATCC-3'	This study
PpkA^{D161AN166A}-2		5'-GGATATGTGCATTGC**GCC**GTCACGCCAGCC**GCT**ATTCTGCTGGCGGATG-3'	This study

a: Restriction enzyme sites are underlined, and mutated sequences are indicated by bold type.

References

1. Ma LS, Lin JS, Lai EM (2009) An IcmF family protein, $ImpL_M$, is an integral inner membrane protein interacting with $ImpK_L$, and its walker a motif is required for type VI secretion system-mediated Hcp secretion in *Agrobacterium tumefaciens*. J Bacteriol 191: 4316-4329.
2. Lin JS, Ma LS, Lai EM (2013) Systematic Dissection of the *Agrobacterium* Type VI Secretion System Reveals Machinery and Secreted Components for Subcomplex Formation. PLoS One 8: e67647.
