# Supplementary material for: Fha Interaction with Phosphothreonine of TssL Activates Type VI Secretion in Agrobacterium tumefaciens
Source: PLoS Pathog. 2014 Mar 13;10(3):e1003991. doi: 10.1371/journal.ppat.1003991 (PMC3953482; doi:10.1371/journal.ppat.1003991)
Supplement: Information S1 — Supporting Information. (PDF) [file ppat.1003991.s014.pdf]

Information S1

Plasmid construction and generation of in-frame deletion or amino acid substitution(s) mutants. The plasmid pJQ200KS-Δ*t6* was created by ligating the *Bam*HI/*Xma*I-digested PCR product 1 (~500 bp DNA fragment downstream of the *ppkA* open reading frame [ORF]) and the *BamH*I/*Xba*I-digested PCR product 2 (~500 bp DNA fragment downstream of the *atu4352* ORF), which were amplified with primers in Table S2, into *Xba*I/*Xma*I sites of pJQ200KS [1] and used to generate the entire T6SS gene cluster deletion mutant Δ*t6* (EML3685).

The remaining pJQ200KS derivatives harboring the *fha*, *tssL*, and *ppkA* genes with amino acid deletion or substitutions, including their respective upstream and downstream regions, were used for double crossover. The PCR products were amplified with primers described in Table S2 and digested by *Xba*I/*Xma*I and cloned into the same sites of pJQ200KS, thus resulting in the plasmids pJQ200KS-*fha*$^{\Delta FHA}$, pJQ200KS-*fha*^{R30A}, pJQ200KS-*fha*^{S46A}, pJQ200KS-*fha*^{R30AS46A}, pJQ200KS-*tssL*^{T14A}, pJQ200KS-*tssL*^{T14D}, pJQ200KS-*tssL*^{T14E}, and pJQ200KS-*ppkA*^{D161AN166A}. The resulting strains were confirmed by PCR and designated as EML3679 (*fha*$^{\Delta FHA}$), EML3694 (*fha*^{R30A}), EML3696 (*fha*^{S46A}), EML3698 (*fha*^{R30AS46A}), EML3709 (*tssL*^{T14A}), EML3855 (*tssL*^{T14D}), EML3858 (*tssL*^{T14E}), and EML4163 (*ppkA*^{D161AN166A}), respectively.

For complementation, the gene of interest containing its ribosomal-binding sequence (RBS) and ORF was cloned to be driven by a *lac* promoter on the broad host range vector pRL662 [2]. The PCR products of *ppkA* and *tagF-pppA* genes were digested by *Xho*I/*Xba*I and *BamH*I/*Xba*I, respectively, and cloned into the same sites of pRL662, which resulted in the plasmids pPpkA and pTagF-PppA.

The remaining *tssL* genes with amino acid substitution or His-tag or Strep-tag

were PCR-amplified with primers in Table S2, digested by *Xho*I/*Xba*I and cloned into the same sites of pRL662, thus resulting in the plasmids pTssL^{T14A}, pTssL-His, pTssL^{T14A}-His, pTssL-Strep, and pTssL^{T14A}-Strep.

The expression vector pET22b(+) was used to overexpress proteins driven by the T7 promoter via isopropyl-beta-D-thiogalactoside (IPTG) induction in *E. coli* BL21 (DE3). Each ORF (without stop codon) encoding wild-type Fha7-267, Fha7-267 with R30A and S46A substitutions, and Fha7-309 was PCR-amplified with primers in Table S2, digested by *Nde*I/*Xho*I and cloned into the same sites of pET22b(+), thus resulting in the plasmids pET-Fha7-267^{WT}-His, pET-Fha7-267^{R30AS46A}-His, and pET-Fha7-309^{WT}-His.

Hcp and Atu4347 secretion assay on agar plate. To perform the Hcp and Atu4347 secretion from *A. tumefaciens* grown on agar plate, *A. tumefaciens* cells were grown in liquid 523 medium for 16 hr at 28°C. Cells were harvested and OD_{600} was adjusted to 1, with 100 µl cell suspension plated on AB-MES medium (pH 5.5) containing 1.5% bacteriological agar (Amresco) and grown at 25°C for 24 hr. Cells were collected by adding 5 ml AB-MES medium (pH 5.5) and the cell suspension was centrifuged at 10,000 x g for 15 min at 4°C; the resulting supernatant was concentrated by TCA for Hcp and Atu4347 secretion assay as described [3,4].

References

1. Quandt J, Hynes MF (1993) Versatile suicide vectors which allow direct selection for gene replacement in gram-negative bacteria. Gene 127: 15-21.
2. Vergunst AC, Schrammeijer B, den Dulk-Ras A, de Vlaam CM, Regensburg-Tuink TJ, et al. (2000) VirB/D4-dependent protein translocation from *Agrobacterium* into plant cells. Science 290: 979-982.
3. Wu HY, Chung PC, Shih HW, Wen SR, Lai EM (2008) Secretome analysis uncovers an Hcp-family protein secreted via a type VI secretion system in *Agrobacterium tumefaciens*. J Bacteriol 190: 2841-2850.

4. Ma LS, Lin JS, Lai EM (2009) An IcmF family protein, $ImpL_M$, is an integral inner membrane protein interacting with $ImpK_L$, and its walker a motif is required for type VI secretion system-mediated Hcp secretion in *Agrobacterium tumefaciens*. J Bacteriol 191: 4316-4329.
